# Constructing and Using Cell Type Populations of the Human Reference Atlas

**DOI:** 10.1101/2025.08.14.670406

**Published:** 2025-08-20

**Authors:** Andreas Bueckle, Bruce W. Herr, Lu Chen, Daniel Bolin, Danial Qaurooni, Michael Ginda, Yashvardhan Jain, Aleix Puig-Barbe, Kristin Ardlie, Fusheng Wang, Katy Börner

**Affiliations:** 1 Department of Intelligent Systems Engineering, Luddy School of Informatics, Computing, and Engineering, Indiana University, Bloomington, IN, 47408, USA; 2 Department of Computer Science and Department of Biomedical Informatics, Stony Brook University, Stony Brook, NY, 11794, USA; 3 European Molecular Biology Laboratory-European Bioinformatics Institute, Wellcome Genome Campus, Hinxton, Cambridge CB10 1SD, UK; 4 Broad Institute, Cambridge, MA, 02142, USA

## Abstract

The human body contains ~27–36 trillion cells of up to 10,000 cell types (CTs) within a volume of ~62–120 liters (males) and 52–89 liters (females). The Human Reference Atlas (HRA) v2.3 provides a quantitative 3D framework of CTs across 73 reference organs and 1,283 3D anatomical structures (ASs). The HRA Cell Type Population (HRApop) effort quantifies CTs per AS using high-quality single-cell (sc) data processed through scalable, reproducible workflows and cell type annotation (CTann) tools. HRApop v1.0 includes reference CT populations for 73 ASs (112 when sex-specific) using 662 datasets spatially registered to 230 locations across 17 organs (31 when sex-specific). For 558 sc-transcriptomics datasets (11,042,750 cells), CTs and biomarker expression were computed using Azimuth, CellTypist, and popV. To test generalizability, 104 sc-proteomics datasets (16,576,863 cells) were integrated. In total, HRApop includes 27,619,613 cells. HRApop can be used to predict (1) CT populations for 3D volumes in the human body and (2) the spatial origin of a tissue block, given a CT population. Data and code are at cns-iu.github.io/hra-cell-type-populations-supporting-information.

## Background & Summary

### The Need.

The volume^1^ of the adult human body is estimated to range from 62–120 liters (0.062–0.120 m^3^) in males with 36 or 37^2^ trillion cells to 52–89 liters (0.052–0.089 m^3^) in females with 27 trillion cells^3^. There is no consensus on the number of CTs within the human body. Estimates range from 400 major CTs^3–6^ to 3,358^7^ total CTs, and depend on the criteria used to determine what constitutes a CT (see the Methods section for a more detailed discussion). Efforts like the Human BioMolecular Atlas Program (HuBMAP)^8,9^, Human Cell Atlas (HCA)^10–12^, and many of the 20+ other atlas efforts contributing to the HRA endeavor aim to provide clarity based on high-quality experimental data collection and analysis.

Most atlas projects aim to capture the number and type of cells per AS together with biomarker expression values—based on expert knowledge or experimental data. For example, the Blue Brain Cell Atlas^13^ (bbp.epfl.ch/nexus/cell-atlas) features 3D data for the mouse brain with CT populations programmatically placed inside 737 brain regions defined in the Allen Mouse Brain Atlas^14^; different ASs; regions and their CT populations can be toggled on and off, and the color hue can be set to encode cell regions, types, or density; cell counts for neurons and glia (with confidence values) are also displayed. The Genotype-Tissue Expression (GTEx) sc-portal^15^ (gtexportal.org/home/singleCellOverviewPage) features CT populations from 25 tissue blocks in eight organs from 16 donors, and the Chan Zuckerberg Initiative (CZI) CELLxGENE (CxG) Portal^16^ (cellxgene.cziscience.com/datasets) features CT populations for 325 human, organ-level ASs but without visual representations of these structures. At the functional tissue unit (FTU) level^17^, Anatomograms^18,19^ developed by the Gene Expression Team at the European Molecular Biology Laboratory-European Bioinformatics Institute (EMBL-EBI) in collaboration with the Wellcome Sanger Institute; the Kidney Precision Medicine Project (KPMP)^20,21^ Explorer (atlas.kpmp.org/explorer); and the HRA FTU Explorer (apps.humanatlas.io/ftu-explorer) feature 2D medical illustrations with associated CT populations.

Given that life unfolds in 3D, there is a strong interest to capture CT populations for ASs in the human body in 3D. The HRA v2.3 features the 3D shape, size, location, and rotation of 1,283 3D ASs, which can be explored at humanatlas.io/3d-reference-library; each 3D AS belongs to one of 73 organs, also called 3D reference objects (see Box 1 in a related publication^22^). As new organ expert teams join the HRA effort, new 3D structures for male and female are added. Data from sc-portals such as GTEx, CxG, KPMP (atlas.kpmp.org), the HuBMAP Data Portal (portal.hubmapconsortium.org), or the Cellular Senescence Network (SenNet) Data Portal^23^ (data.sennetconsortium.org/search) can be spatially registered into the 3D reference objects using the web-deployed HRA Registration User Interface (RUI, apps.humanatlas.io/rui)^24,25^ and guided by standard operating procedures (SOPs)^26–28^.

Ultimately, HRApop can serve as a healthy reference to elucidate mechanisms underlying cellular interactions and, as a result, point to changes in the body that signify disease progression, which may facilitate advancements in basic discovery and lead to new therapeutic strategies.

### The Challenge.

Computation of CT populations for the many different ASs in the human body requires both a 3D registration and reproducible dissociation protocols that allow isolating single cells (or single nuclei) from tissue in support of sc-transcriptomics analyses^29–31^ or high quality cell segmentation for sc-proteomics spatial data^32,33^. In both cases, CTann tools are needed to assign a CT to each cell. Most sc-segmentation and annotation tools are organ specific. However, human organs are large (an average human kidney is about 10–12 cm long, 5–7 cm wide, and 3 cm thick and is estimated to contain about 110 billion cells^3^). Organs have many different internal ASs with vastly different CT populations that serve diverse physiological functions. The HRA^34,35^ used the Visible Human Project data^35–38^ and the expertise of medical illustrators to segment 1,283 3D ASs in 37 vital organs; given tissue data that is spatially registered into these 3D AS using the RUI^24^ plus sc-dissociation/segmentations and annotations^32,39–47^, the number of cells per CT can be computed for specific ASs, i.e., 15 AS within the male lung. To compute HRApop at AS (not organ level), 1,000s of datasets from four different portals are downloaded, donor metadata is harmonized, and multiple CTann tools are run for each sc-transcriptomics dataset. This paper explains exactly how dataset, extraction site, and AS-specific CT populations and biomarker expression values are computed at scale and how they are used to predict CT populations for 617 tissue blocks and spatial origin for 5,453 datasets in support of quantifying cross-AS and cross-individual variation of cellular phenotypes; how open HRApop v1.0 data is published via the HRA; and what known limitations and next steps exist for the work presented.

**Fig. 1.**
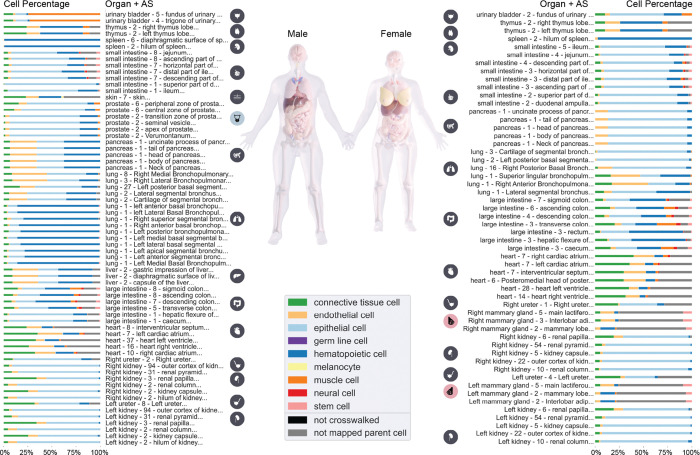
CT populations for unique ASs across male and female in HRApop v1.0. Stacked bar graphs of the percentage of CT identified in ASs, aggregated to higher level CTs from CL, see listing on GitHub^55^, shown for male (left) and female (right, placenta was omitted). Male-only organ (prostate) icon is rendered in blue while icons for female-only organs are rendered in pink (mammary gland left and right).

Fig. 1 shows the 112 ASs of the male (left) and female (right) reference body for which spatially registered sc-transcriptomics data exist. For each AS, the organ name, number of datasets, and AS name plus a bar graph with the percentage of major CTs, so-called AS Cell Type Populations (ASpop, see Box 1), are shown. Only sc-transcriptomics data is shown and three CTann tools were used to annotate cells: Azimuth^48^, CellTypist^49,50^, and/or popV^51^; with a preference for Azimuth annotation over CellTypist over popV^51^, see Methods section for details. Crosswalking to Cell Ontology (CL, www.ebi.ac.uk/ols4/ontologies/cl)^52–54^ and aggregation to higher-level CTs is also described in detail in the Methods section. For the left and right mammary gland, two CTs that cannot be mapped to a high-level CT make up ~70% of the CTs across ASs. As a result, the stacked bar graphs for these are mostly grey (luminal epithelial cell of mammary gland [purl.obolibrary.org/obo/CL_0002326] with ~53% and fibroblast of breast [purl.obolibrary.org/obo/CL_4006000] with ~16%).

### The Opportunity.

**Fig. 2.**
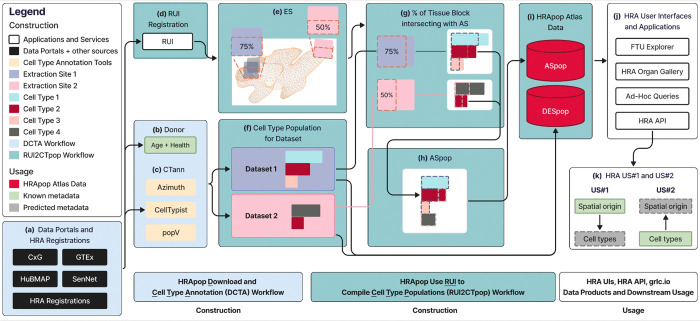
Construction and usage of HRApop. **(a)** Data is ingested from four data portals or from HRA Registrations^57^. **(b)** Donor information is extracted from dataset metadata or the extraction site (if from HRA Registrations). **(d)** CT populations are computed using CTann tools if possible. **(a)-(c)** is handled by the **DCTA Workflow**. **(d)** RUI-assigned extraction sites for each dataset are identified. If a dataset has an extraction site, **(e)** intersection percentages (see Box 1) of the extraction site with ASs can be computed. **(f)** CT populations for corresponding datasets obtained via one or more CTann tools can be combined with **(g)** intersection percentages between extraction site and AS, resulting in **(h)** ASpop, which, together with DESpop, are then published as **(i)** HRApop Atlas Data. **(d)-(i)** is handled by the **RUI2CTpop Workflow**. **(j)** illustrates usage of HRApop Atlas Data by HRA applications. **(k)** shows usage for HRA US#1 and US#2.

Using these ASpop based on all the tissue blocks registered into an AS with the RUI, it becomes possible to (1) predict CT populations for 3D volumes in the human body that have sufficient data and (2) predict 3D origin volumes for a tissue block with known ASpop (see Fig. 2.K and details in the Usage Notes section). In addition, other HRA queries can be run, e.g., a query to retrieve all AS-CT combinations, with sex, tool, CT, and cell percentage is available in the Usage Notes section, which also points to Python example code for using this query to get ASpop as well as Dataset and Extraction Site Cell Type Populations (DESpop) from the HRA Knowledge Graph (KG)^22^.

## Results

The HRApop effort combines (1) CT populations from sc-transcriptomics and sc-proteomics data made with CTann tools (or via user-assigned CT label), (2) donor metadata, and (3) 3D extraction sites made via the RUI. This involves running two scalable workflows that were optimized to effectively deal with the continuously growing number of datasets. Over the last three years, the processes used to download data, perform cell type annotations, and compute CT populations per dataset (called **DCTA Workflow**) and the workflow that takes CT populations, donor metadata, and 3D extraction sites via the RUI, to compile Cell Type Populations for ASs, datasets, and extraction sites (called **RUI2CTpop Workflow**). These are then published as graphs in the HRA KG^22^ for usage in various HRA applications and the HRA Application Programming (API, apps.humanatlas.io/api). Note that CT populations record the number of cells per CT not just for ASs but also for datasets and extraction sites as well as the top-10 biomarkers per CT (datasets only) and their mean expression values (see Methods section on how these are computed).

Subsequently, this paper details how these two workflows are used in HRApop construction; describe multiple HRApop usages; and list counts for HRApop v1.0, including raw datasets, extraction sites, experimental data, and data products in the HRA KG, following LOD principles^59^ and enabling FAIR^60^ data access; then, it details a generalization to sc-proteomics data. This section concludes with limitations and outlook for future work on improving the HRApop Atlas Data presented. In the remainder of the paper, the Methods section details implementation of the DCTA and RUI2CTpop Workflows. The Data Records section details where data is made available to users. The Technical Validation section elaborates on (1) the prevalence of different CTs inside not just the organs but also the ASs of the human body and (2) the number of datasets per organ and AS. Finally, the Usage Notes describe how data access is facilitated.

### Construction

**HRApop** is constructed with two automated workflows:

1. The **D****ownload and** **C****ell** **T****ype** **A****nnotation (DCTA) Workflow** (see GitHub^61^ and Box 1) uses scalable, open-source software to programmatically download and annotate H5AD files from four portals: HuBMAP, SenNet, GTEx, and CxG. Cells in these H5AD files are then annotated with containerized CTann tools kept in the **HRApop CTann Tool Containers** (see **GitHub**^62^ and Box 1); next, the DCTA Workflow crosswalks the resulting CTann labels to CL^52^ or Provisional Cell Ontology (PCL)^63^, see Methods section for details, then compiles donor metadata from each dataset and finally makes the result available as a set of CSV files (for metadata) and JSON files (for CT populations). These can then be used as input for the next workflow.
2. The **RUI** **to Compile** **CTpop** **(RUI2CTpop) Workflow** (see GitHub^64^ and Box 1) then computes CT populations for ASs, datasets, and extraction sites. It first identifies all datasets that can be used to construct HRApop based on four Criteria (C1–4, see Box 1):
  - **C1:** The dataset has a 3D extraction site by registration with the RUI (see Methods), i.e., the spatial position, rotation, and size of the tissue block (from which the dataset was derived) within the HRA reference system is known and AS tags exist.
  - **C2:** The dataset has a CT population (see Box 1), i.e., number of cells per CT. For sc-transcriptomics data, this means having an associated H5AD file with a cell by gene matrix that can be annotated via Azimuth^48^, CellTypist^49,50^, and/or popV^51^; for sc-proteomics data, CT populations are obtained via manual segmentation and annotation workflows.
  - **C3:** The dataset is of high quality, i.e., it was downloaded from a data portal with built-in quality assurance/quality control (QA/QC) or is associated with a peer-reviewed publication.
  - **C4:** The dataset has complete donor metadata, is from a healthy tissue sample, and has an age value greater than 18. That is, the data is from a healthy adult human.

**C1** and **C2** are enforced to enable usage of HRApop Atlas Data for HRA User Story (US)^34^ #1 (predict CT populations) and US#2 (predict spatial origin of tissue samples). **C3** ensures that atlas data comes from reliable, scientifically backed sources. **C4** is in support of constructing a healthy reference for HRA US#3 (compare reference tissue with aging/diseased tissue). In the future, a disease reference will be built with adjusted criteria.

For datasets that meet **C1–4**, RUI2CTpop outputs the **HRApop Atlas Data** (also called “**HRApop Data Used in Atlas Construction,**” see Box 1), a collection of data products that describe high-quality ASpop and DESpop for the HRA.

Datasets that do not meet all four criteria are disregarded, except: Datasets that **fulfill C3–4** and either **C1** or **C2** are added to the **HRApop Prediction Data**, for which HRApop can be used to predict either an extraction site or a CT population (see Box 1 and Outlook section for details as well as Table S1 for download links).

Fig. 2 shows construction and usage of HRApop, from the data download on the left to the publication and usage of HRApop Atlas Data on the right. It provides an overview of data download and CTann runs via the DCTA Workflow as well as ASpop and DESpop computation via the RUI2CTpop Workflow, see details in the Methods section.

First, in the **DCTA Workflow** (blue), datasets represented as H5AD files are programmatically downloaded from the four data portals (Fig. 2.a) alongside donor metadata (Fig. 2.b). Before download, non-human and diseased data is filtered out. Then, each dataset is annotated using all applicable CTann tools (Fig. 2.c), resulting in CT populations if the dataset originates from a supported organ (Fig. 2.f). Only if a dataset meets all Criteria C1–4 (see Box 1), then it is used for HRApop Atlas construction.

Next, in the **RUI2CTpop Workflow** (green), datasets are matched against all existing RUI extraction sites by their ID (Fig. 2.d); the extraction sites hold metadata on organ sex and laterality (left or right) and can come from either an API (such as for HuBMAP or SenNet, see Methods section) or the static HRA Registrations^57^ (see Table S1), a manually curated collection of extraction sites.

For each HRApop Atlas Dataset, i.e., that has been determined to fulfil Criteria C1–4 (see Box 1), the 3D ASs that its extraction site collides with are determined via mesh-based collision detection (Fig. 2.e, see also Box 1). The example shown is the renal pyramid (purl.obolibrary.org/obo/UBERON_0004200) of the left, male kidney (lod.humanatlas.io/ref-organ/kidney-male-left/latest) with two hypothetical tissue blocks colliding with it at 75% and 50% of the total volume of the extraction site. The number and percentage of CTs that should be in the colliding AS based on the intersection percentage (see Box 1) of the extraction site are computed (see Fig. 2.g). This is performed for every extraction site that intersects with the AS. The result is an ASpop containing the unique and shared CTs contributed by each colliding extraction site (see Fig. 2.h and details in Methods).

In cases where there exists just one tissue block (see definition in related HRA publication^34^) that is registered into exactly one 3D AS, this AS is assumed to have the CT population and biomarker expression levels of that tissue block; plus, confidence will be low as only one dataset exists for that AS. Typically, many tissue blocks from different donor demographics (e.g., age, ethnicity) exist per male/female-specific ASs. Often, research teams carefully sample from the very same RUI extraction site (see definition in a related publication^34^) so hundreds of datasets have the very same extraction site. In some cases, a tissue block is registered with more than one AS; here, CT populations are split using the percentage of mesh-based collisions detected (see Box 1) to associate cells to ASs. Finally, the ASpop and the DESpop used to generate the ASpop are published as two separate Resource Description Framework (RDF, www.w3.org/RDF) graphs (see Fig. 2.i) via the HRA KG^22^ at lod.humanatlas.io/graph/hra-pop/latest in support of Linked Open Data (LOD)^59^ principles.

### Usage

Finally, the data products generated as part of this RUI2CTpop Workflow are then used in various HRA UIs and the HRA API (Fig. 2.j), which then makes them available for usage in HRA US#1 and US#2 (Fig. 2.k).

HRA KG queries can be run to support diverse HRA applications (see Fig. 2.j). Examples are the HRA FTU Explorer (apps.humanatlas.io/ftu-explorer), the HRA Organ Gallery in virtual reality (VR)^65,66^, and ad-hoc queries, such as one that provides an overview of all AS-CT combinations, including sex, tool, cell count, and cell percentage, available at apps.humanatlas.io/api/grlc/hra-pop.html#get-/cell_types_in_anatomical_structurescts_per_as. Via the HRA API (apps.humanatlas.io/api), the ASpop and DESpop are then used for the Cell Population Predictor and the Tissue Origin Predictor in support of HRA User Stories^34^ #1 and #2 (see Fig. 2.k and details in the Usage Notes section).

An exemplary ASpop from the kidney and an exemplary snippet of the DESpop are available on the companion website at cns-iu.github.io/hra-cell-type-populations-supporting-information#exemplary-cell-summaries. A complete listing of all data and code for construction and usage of HRApop is provided in Table S1. An overview of all HRA applications that use **HRApop Atlas Data** is provided in Table S2.

The Usage Notes section introduces a pointer to a series of Jupyter Notebooks that allow users to run the DCTA Workflow on a local H5AD-file, details API queries to access HRApop data programmatically, and details two no-code demo applications (**Cell Population Predictor** and **Tissue Origin Predictor**) provide UIs to implement US#1 and US#2, respectively.

### Counts for HRApop v1.0

#### Raw Datasets

On June 16, 2025, the RUI2CTpop Workflow was run to compile HRApop v1.0. It downloaded 16,293 datasets with 57,911,931 cells from the four sc-portals, which were then sent through a filtering process (see Methods section for details). Additionally, as of August 2025, 1,746 tissue blocks from 1,327 different 3D cuboid extraction sites exist across 30 organs and they link to 6,378 tissue datasets.

558 of the 662 datasets in the HRApop Atlas Data are sc-transcriptomics datasets that were annotated using one or more of three major CTann tools (Azimuth^48^, CellTypist^49,50^, popV^51^) covering 11,042,750 unique cells in a total 3D volume of ~12.05 liters (dm^3^) with partially intersecting extraction sites in 73 unique ASs in 17 organs (112 ASs and 31 organs if male and female are counted separately). The datasets come from 230 3D extraction sites that cover 54 3D ASs across 17 organs. While the HRApop Atlas focuses on these 558 sc-transcriptomics datasets in 17 organs, the method is generalizable to sc-proteomics (see Generalization to Spatial Data section) and to all organs.

Table 1 provides counts of datasets and cells in the HRApop Atlas Data, split by sex, consortium, number of datasets, number of cells, and modality. HuBMAP, SenNet, and GTEx have their own portals. HCA and NHLBI/LungMap^67,68^ datasets all come from the CxG Portal. Detailed counts for the HRApop Atlas from the RUI2CTpop Workflow are provided in Table S3.

#### HRA Registrations and Extraction Sites via APIs

For all extraction sites, mesh-based collision detection (see Box 1) is used to compute the intersection percentage (see Box 1) with 3D ASs, which enables the construction and aggregation of CT populations for these 3D ASs. As a result, extraction sites and ASs in the HRA are enriched with the number of cells per CT from high-quality data. Where possible, for all datasets, extraction sites, and ASs, HRApop provides CT populations from every tool that can annotate the dataset. All extraction sites used in the HRApop Atlas Data are shown in the HRApop-focused Exploration User Interface^24^ (EUI) at cns-iu.github.io/hra-cell-type-populations-supporting-information/eui.html. Each extraction site in the HRApop Atlas also has a corridor (see Box 1 and Methods section). By working closely with authors of published, high-quality datasets, as well as tissue providers in HuBMAP and SenNet, RUI is used to generate extraction sites for a growing number of organs and ASs.

Table 2 presents counts for datasets, extraction sites, ASs, and organs based on input data for RUI2CTpop Workflow and the HRApop Atlas.

#### Experimental Data

**Fig. 3.**
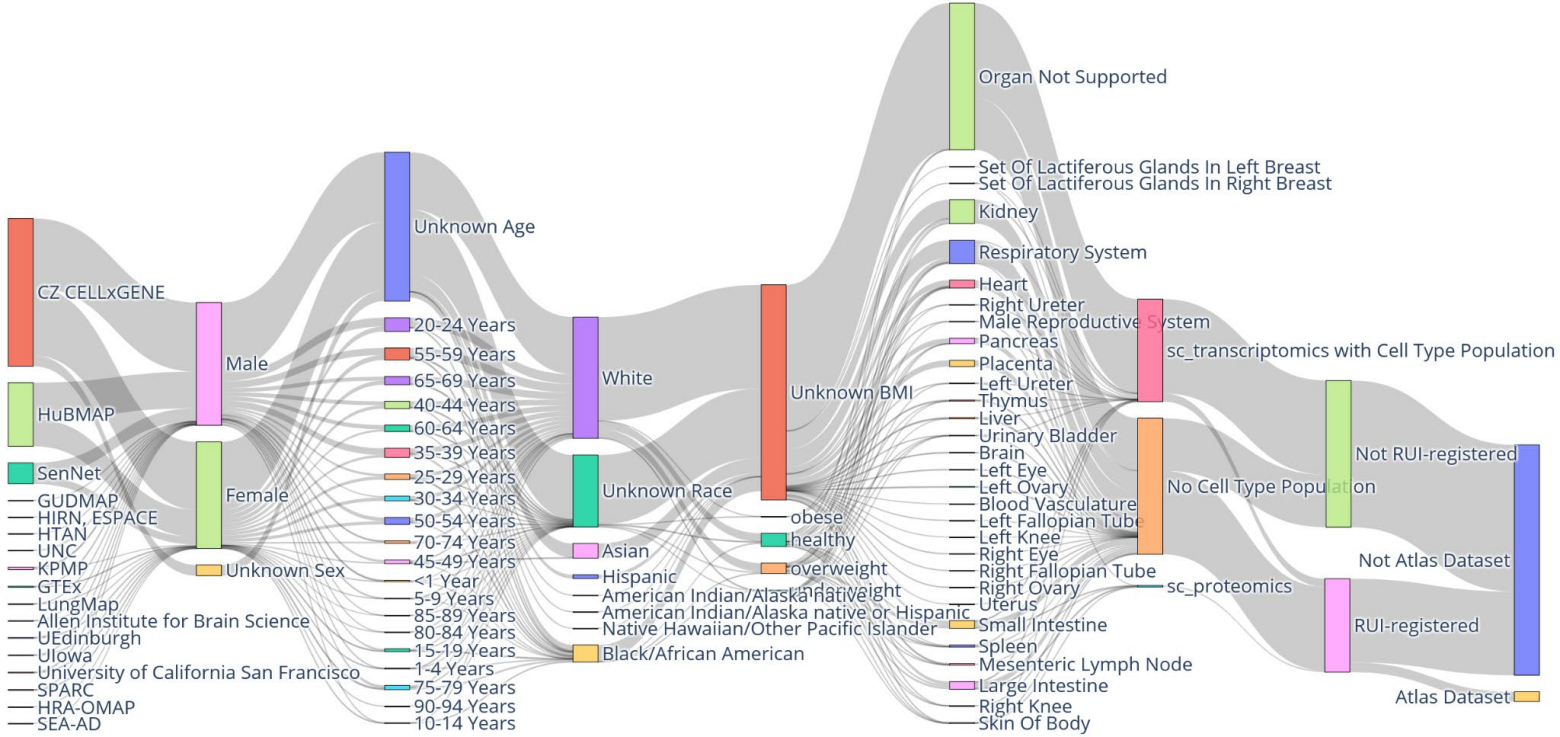
Sankey diagram of all input data for the input for RUI2CTpop Workflow. Note the Atlas Data node in the bottom right corner, which comprises the 662 HRApop Atlas Datasets in HRApop v1.0.

The Sankey diagram in Fig. 3 provides a high-level overview of the input data for the input for RUI2CTpop Workflow along several axes, see below. It can be explored interactively at cns-iu.github.io/hra-cell-type-populations-supporting-information/sankey_universe_plotly.html. A version showing only HRApop Atlas Data is shown in Fig. S1, with an interactive version available at cns-iu.github.io/hra-cell-type-populations-supporting-information/sankey_atlas_plotly.html.

The Sankey diagram has 9 vertical axes that represent:

##### Portal/source:

Identifies the effort where the data originates. H5AD files were downloaded from HuBMAP, SenNet, GTEx, and CxG. The majority of datasets come from CxG. All other portals/sources were derived from extraction sites. More information about data portals is provided in the Methods section.

##### Donor sex, donor age, donor race, donor body mass index (BMI):

Describe clinical metadata for the human specimens from which the data was retrieved. Where available, donor age is provided as an integer by HuBMAP and SenNet but as string by CxG (e.g., “61-year-old human stage”); as a result, string literal age values for CxG data were parsed as a number where possible. To enable visualization in a Sankey diagram, all age values were aggregated into bins of five years. For race, the same categories were used as on the HuBMAP Data Portal (portal.hubmapconsortium.org) as of the writing of this paper. BMI values were mapped to categories by brackets defined by the Centers for Disease Control on www.cdc.gov/bmi/adult-calculator/bmi-categories.html.

##### Organ:

Indicates the organ of origin, where “Organ Not Supported” means that there is no matching 3D reference object in the HRA (such as for blood).

##### CT population:

Means that a dataset has either (1) one or multiple CT populations from one or multiple CTann tools (“sc_transcriptomics with Cell Type Population”), (2) no CT population if no CTann tool exists for the dataset (“No Cell Type Population”), or (3) a CT population via sc-proteomics as a generalization of the HRApop workflow (“sc_proteomics with Cell Type Population”).

##### Extraction site:

Indicates whether a dataset has an extraction site via RUI registration or not.

##### HRApop Atlas Data:

Indicates whether the dataset is part of the HRApop Atlas or not.

#### HRApop in HRA KG

Five data products of HRApop v1.0 are available on Zenodo^69^ (see Data Records section and Table S1 for details) and three are available via the HRA KG (lod.humanatlas.io/graph/hra-pop/v1.0):

**DESpop** (called atlas-enriched-dataset-graph.jsonld on the HRA KG) list CTs with top-10 biomarkers for 662 datasets (558 sc-transcriptomics, 104 sc-proteomics) as well as CTs, mesh collisions, and 230 corridors for 230 3D extraction sites.

**ASpop** (called atlas-as-cell-summaries.jsonld on the HRA KG) list counts and percentage of CTs for all 73 ASs in 17 organs covered in HRApop v1.0. Additional counts for DESpop and ASpop are provided in Table 2 above.

**Corridor GLB files** (named after the extraction site on which they are based, e.g., corridors/00087766-0287-467c-9060-b52773db3dce.glb) describe corridors, see Methods section. The HRA KG makes 1,189 available, including the 230 corridors for the 230 extraction sites in DESpop, plus 959 corridors for extraction sites not in the HRApop Atlas. Their total size is 202 MB. When compressed and placed on Zenodo^69^ as a ZIP file, they are 99.8 MB large.

### Generalization to Spatial Data

In HRApop v1.0, 104 sc-proteomics datasets contribute 16,576,863 cells, which brings the total number of cells to 27,619,613. They are associated with high-quality publications^34,70–74^ and use protein and antibody-based modalities such as Cyclic Immunofluorescence (CyCIF)^75^, Cell DIVE^76–78^, and co-detection by indexing (CODEX)^79^ to identify proteins and quantify their expressions in a tissue in situ; there are plans to integrate iterative bleaching extends multiplexity (IBEX)^80,81^ datasets in the future. While this paper does not focus on sc-proteomics data, it presents a curated collection of sc-proteomics datasets as a generalized use case for HRApop. Spatial proteomics has received significant interest from the scientific community in recent years^82^, which has led to increased high-quality data generation. If integrated in HRApop, it enables creating CT populations with a preserved spatial context for each cell (which is lost in sc-transcriptomics datasets, although some recent work has attempted recoveries for specific assay types and organs^47^).

The DCTA Workflow outputs CT populations and metadata for all datasets, both sc-transcriptomics and scproteomics. While sc-transcriptomics datasets are run through at least one CTann tool, sc-proteomics datasets have CTs assigned by their expert providers, who share CT populations in the form of CSV files, see code in Table S1. The DCTA Workflow systematically goes through a list of sc-proteomics datasets (available on GitHub^83^) and transforms the files with nodes for each cell in each dataset (CSV) into a CT population (JSON) as an input for the RUI2CTpop Workflow (see GitHub^84^). In the future, the DCTA Workflow will be extended to (1) handle other generalized use cases that cannot be annotated with CTann tools and (2) run more CTann tools over new and existing HRApop datasets for additional CT populations.

To make crosswalks for sc-proteomics data, CT labels were shared by contributors of high-quality datasets; crosswalks for each dataset were manually created for that publication effort and made available at lod.humanatlas.io/ctann/vccf/latest. Unmapped CTs are on GitHub^85^.

## Limitations

HRApop v1.0 comes with a number of limitations:

### Dataset duplication across data portals:

Some datasets (e.g., by KPMP) are available via multiple data portals (e.g., HuBMAP and CxG). However, no dataset should be used twice for HRApop construction. Data duplication detection is difficult as different versions of the data might exist and different metadata might be associated with each version. For example, there might be a dataset submitted with the very first paper submission and linked to a preprint on a preprint server, a slightly expanded dataset associated with a revised and later preprint version, and a final version of the dataset linked to a peer-reviewed published paper; paper title and authors might also change in the process making deduplication challenging.

### CTann tools are trained on underspecified data:

CTann tools are trained on high-quality reference datasets that might not be separated by sex or even donor and for which RUI registration information is not available; i.e., it is unknown from which of the diverse ASs within an organ a tissue that was used to train the CTann tool originated. HRApop, however, utilizes existing CTann tools to compile ASpop that are specific to the diverse ASs and tailored to the male and female human body. As more RUI registered sc-datasets become available, CTann tool developers might like to use this additional tissue origin information to optimize CTann.

### Missing assay type information needed for batch correction:

At present, the ds-graph HRA Digital Object type^22^, which captures datasets and their extraction sites plus donor metadata, only contains assay type metadata as free text string rather than not ontology terms (e.g., 0× 3’ v1–3, 10x scATAC-seq, MERFISH, Smart-seq, all listed on the CxG portal). HRA KG remodeling is underway to provide look-up tables for assay types to ontology terms for enabling more systematic queries.

### Intersecting 3D reference objects:

The 1,283 3D ASs are supposed to have no overlap with each so that CT populations are specific to one, not multiple intersecting ASs. However, in HRA v2.3, 18 ASs in 17 organs (e.g., two in the female heart) have intersections with each other and also have extraction sites in them (labeled “TB3” in Fig. S2). The AS-AS pairs with the most (here three) extraction sites that collide with both ASs are the ‘outer cortex of kidney’ and the ‘renal pyramid A’ in the male, left kidney and the ‘kidney capsule’ and the ‘outer cortex of kidney’ in the female, left kidney. Intersections will be corrected and tissue blocks will be re-registered for an upcoming HRA release.

### Limited coverage of organs and ASs:

The HRApop effort aims to enrich all 1,283 3D ASs in 73 3D reference objects of the entire HRA v2.3 with CT populations from high-quality datasets. However, at present, only 73 3D ASs (112 if male and female are counted separately) in 17 organs (31 if male and female are counted separately) have data to compute AS-specific CT populations. The Technical Validation section expands on current HRApop coverage of organs and ASs.

### Prediction accuracy:

Due to the limited number of spatially registered datasets in HRApop, the ability to use HRApop to predict CT populations in support of HRA US#1 and the spatial origin of tissue samples in support of HRA US#2 is limited. About 1,000 more datasets with more than 50 million cells will be added in the next HRA release, see Outlook section. Moreover, as shown in the Technical Validation section, there exist discrepancies in coverage between male and female organs and CTann tools, where datasets from male donors and specific organs (heart, large/small intestine, lung) are overrepresented.

## Outlook

Regular HRApop releases are planned and scheduled, with data products made available via the HRA KG^22^ (lod.humanatlas.io/graph/hra-pop) though the HRA Content Delivery Network (CDN)^22^ and Zenodo^69^. To improve coverage and quality of HRApop, the following steps are planned:

### Increase number of RUI registered datasets:

As of August 2025, the HuBMAP and SenNet portals list 842 RUI registered datasets that are currently in QA/QC status but will be published soon. Outreach efforts to authors of peer-reviewed, published papers are ongoing to register their data for use in the DCTA Workflow in support of HRApop Atlas Data construction. The HRApop effort will also integrate data from Tabula Sapiens^5^, KPMP, the Helmsley Gut Cell Atlas^86^, and the Deeply Integrated human Single-Cell Omics (DISCO) database^87^, which has a total of 19,717 datasets, out of which 32% of the total data is from a healthy human body, across 159 unique ASs.

### Scale up tissue registration via millitomes:

A millitome^34^ (from Latin *mille*, meaning “thousand,” as in millimeter, and the Greek *temnein*, meaning “to cut”) is a device designed to hold a freshly procured organ and facilitate cutting it into many small tissue blocks of well defined size for usage in sc-analysis and HRA construction. It is used to produce uniformly sized slices or cubes of tissue material that can be registered to 3D reference objects. Using a millitome improves efficiency by enabling consistent, high-throughput sampling.

### Improve generalizability:

The 104 sc-proteomics datasets in HRApop v1.0 were presented as a generalization from sc-transcriptomics datasets. In the future, and in synergy with HRA Vasculature Common Coordinate Framework (VCCF) construction efforts^72,88^, more CODEX^79^ datasets and new modalities, such as the Spatial Multiomics Single-Cell Imaging platform CosMx^89^, will be added to HRApop.

### Increase number of CTann tools used to enable more benchmarking:

Currently, HRApop uses three well-established CTann tools backed by scientific publications describing the methods, results, and validations for each tool. Results are presented as CT populations by CTann tool—users can pick their favorite tool and data or perform comparisons and benchmarks between CTann tools^90^. Future HRA releases will feature additional CTann tools such as FR-Match^91^. This will further enable CTann tool comparisons and benchmarking for the same source data.

### Compare HRApop with similar efforts using the same source data:

Plans exist to compare HRApop with other efforts for computing CT populations. CxG data is heavily used by the sc-community. As a result, benchmarks exist that enable the validation of HRApop CTann results against those obtained by other teams. For example, the CuratedAtlasQueryR package (stemangiola.github.io/CuratedAtlasQueryR) on Bioconductor^92,93^ harmonized 12,981 sc-RNA sequencing samples with 29 million curated cells from 45 anatomical sites.

### Return confidence scores in Cell Population Predictor:

At present, the Cell Population Predictor (see Usage Notes section) returns a CT population for the provided extraction site, including the number, percentage, label, and ontology ID of all CTs separated by CTann tool. Updates to the HRA API are planned to enable this UI to return confidence scores for each CT, like the ones in the cell instances (see Box 1) for sc-transcriptomics datasets in HRApop v1.0 on Zenodo^69^.

### Validate HRApop for HRA US #1 and #2^34^:

**Fig. 4.**
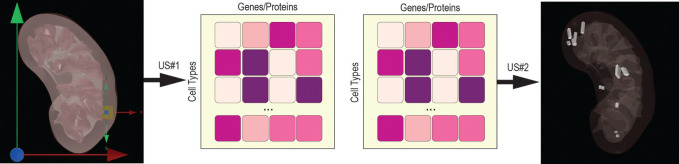
A simplified illustration of HRA US#1 (left) and US#2 (right). Shown for US#1 is a collection of predicted extraction sites.

The Cell Population Predictor and the Tissue Origin Predictor are proof-of-concept UIs with underlying HRA API endpoints that enable no-code predictions, see Fig. 4. Current work aims to validate and improve these predictions for 617 unique extraction sites (US#1) and 5,453 unique datasets (US#2) in the HRApop Prediction Data that is part of HRApop v1.0.

### Decrease run time for HRApop code:

For HRApop v1.0, the DCTA Workflow started on Thu, May 15, 2025, ran for about 10 days, and finished on Sunday, May 25, 2025. It averaged 87.63 dataset annotation runs per hour. Annotations took about 8.59 days to finish. This long runtime is primarily due to the complexity of the annotation algorithms to cover 10s of millions of cells. Targeted optimization of the algorithms and workflows combined with more hardware resources and re-using annotations from prior runs will be required to reduce runtime. Work is underway to save annotations between runs to skip the re-annotation step and work faster. After a 22-day QA phase, the RUI2CTpop Workflow started on June 16 at 5:55:27 PM EDT and finished about four hours later at 10:07:11 PM EDT the same day. A full log is available in Table S1. In the future, the run time for the DCTA Workflow will be decreased by using high performance computing (HPC) in the form of Big Red at Indiana University (kb.iu.edu/d/brcc). Also, the crosswalking will be moved to the RUI2CTpop Workflow, which will decrease runtime and improve modularity of both workflows.

## Methods

### Estimates of Number of CTs in the Human Body

While no consensus exists on the number of CTs within the human body, estimates range from 400 major CTs^3–6^ to 3,358 CTs^7^, depending on the criteria used to determine what constitutes a CT. As an example, in the retina, a major class of retinal neurons is the amacrine cell (purl.obolibrary.org/obo/CL_0000561). This cell can be subdivided in multiple ways. At a broad level, it is often classified into GABAergic (purl.obolibrary.org/obo/CL_4030027) and glycinergic (purl.obolibrary.org/obo/CL_4030028) types (two categories). However, based on morphology, researchers have identified 25 distinct amacrine CTs^94^. At the level of sc-transcriptomics, the Human Retina Cell Atlas (HRCA)^95^ has reported 123 CTs in the retina, including 73 molecularly distinct amacrine CTs. As a result, depending on the resolution—functional class, morphology, or transcriptomic profile—one might count one, two, 25, or 73 different amacrine CTs. In the case of the human brain, sampling more than three million nuclei from approximately 100 dissections across the forebrain, midbrain, and hindbrain, 461 clusters and 3,313 subclusters (granular CTs) organized largely according to developmental origins were identified^96^. Combining CTs in the Human Lung Cell Atlas^97^ and the CellRef atlas from the LungMAP consortium^98^ revealed 68 distinct CTs in the human lung and nasal cavity^90^. As of August 2025, CL^52^ contains 3,358 classes, but this includes many non-human terms as well as grouping classes—i.e., internal nodes in the hierarchy that do not correspond to distinct, terminal CTs. When limiting the count to leaflevel human CTs, the number is on the order of 2,500, but this does not reflect many of the novel CTs that have been recently defined using sc-technologies. Further, about 2,000 CTs^7^ in CL are connected to ASs in the cross-species anatomy ontology Uberon^99,100^ via ‘part of’ relationships. As a result, 400 total CTs might be a clear underestimation. A more plausible estimate is closer to 1,900, though the exact number depends on the criteria used to define and distinguish CTs. Assuming that there are 78 major organs in the human body of varying size and cellular complexity, with most organs averaging between 50 and 120 transcriptomic CTs, aside from the brain with 3,000–5,000, there might be close to 10,000 CTs in adult mammalian organisms, depending on the criteria for distinguishing CTs.

### Data Used

#### Data Portals

##### HuBMAP:

As of August 2025, the HuBMAP Data Portal (portal.hubmapconsortium.org) lists 4,742 public datasets from 27 organs. These datasets are ingested by tissue providers through a UI (ingest.hubmapconsortium.org) where they can enter donors, organs, samples/tissue blocks, tissue sections, etc. Relationships between these entities are organized in a provenance hierarchy where a donor and organ are needed so that tissue samples can be organized based on diverse tissue sample types. APIs enable users to access entities programmatically (docs.hubmapconsortium.org/apis.html). Both published and unpublished datasets exist on the HuBMAP and SenNet portals (see below). Published datasets have been sent through a series of QA/QC processes. Unpublished datasets are only accessible with authentication. Only published datasets are used for HRApop construction.

##### SenNet:

The SenNet Data Portal (data.sennetconsortium.org/search) uses a similar infrastructure as the HuBMAP Data Portal. It features human and murine datasets. As of August 2025, 1,712 human datasets from 903 donors are publicly available. Like for HuBMAP, APIs provide programmatic access to datasets, donors, organs, etc. (docs.sennetconsortium.org/apis).

##### GTEx:

The GTEx Portal (www.gtexportal.org/home/downloads/adult-gtex) hosts the adult GTEx data and resources and provides open access to, e.g., expression quantitative trait loci (eQTLs), and protected access, e.g., to limited donor phenotypes as well as de-identified donor data for sequencing. For GTEx sc-data, one H5AD file with data for all 8 organs plus donor metadata was downloaded (storage.googleapis.com/adult-gtex/single-cell/v9/snrna-seq-data/GTEx_8_tissues_snRNAseq_atlas_071421.public_obs.h5ad).

##### CxG:

The CxG Portal (cellxgene.cziscience.com/collections) provides access to both primary and secondary datasets. Primary datasets contain the raw or minimally processed data while secondary datasets are curated and normalized. The DCTA Workflow retrieves the secondary datasets when possible unless they contain fewer data points than the primary datasets. As of August 2025, there are 1,183 collections with 791 healthy adult donors.

#### Cell Counts

Cell counts represent the unprocessed number of RNA transcripts detected for each gene in each cell. To ensure data integrity and consistency, raw cell counts are used wherever available during CTann. For HuBMAP and SenNet datasets, these are obtained from the “counts” layer of the H5AD file, which contains integer counts per gene and cell, while for GTEx and CxG datasets, raw counts are obtained from the “raw.X” attribute of the H5AD file, which stores original and unnormalized counts for each cell and gene.

#### Crosswalks

To enable comparisons between CTs assigned by different CTann tools (and sc-proteomics data, which use human-assigned CTs), CT labels need to be crosswalked to CTs in the anatomical structures, cell types, plus biomarkers (ASCT+B) tables^35^ using CL or PCL terms. Crosswalks for each CTann tool are curated manually by experts and are published at lod.humanatlas.io/ctann; the underlying HRA Digital Object type is described in a related publication^22^. Since not all CTs have an exact match to a CL term, the Simple Knowledge Organization System (SKOS, www.w3.org/2004/02/skos)^101,102^ is used to indicate if the mapping was done to a term with an exact match (skos:exactMatch) or if they had to be mapped to a more general class (skos:narrowMatch). The crosswalks published in HRA v2.3 link 1,615 annotation labels and 1,909 annotation IDs from Azimuth, CellTypist, popV, and sc-proteomics datasets (author-assigned) to 885 CL labels and 495 CL IDs for 36 organs. 1,923 mappings are exact matches and 683 are narrow matches.

Fig. 1 uses CTann crosswalks to harmonize and compare datasets across portals. The resulting CT typology has 201 low-level CTs that were aggregated into 9 high-level CTs—a subset of broad terms curated by CL editors that covers the majority of CTs with minimal overlap. Additional mappings exist to 19 out of 24 medium-level, more granular CTs—a subset of terms that, while still broad, provides a more detailed classification—via CL IDs and labels; this mapping is available in Table S1. The mapping applied in Fig. 1 uses the 9 CTs belonging to the upper slim of high-level CTs directly from CL (see GitHub^103^); note that CTs from PCL are included in this slim as well. 26 CTs were classified under more than one category in CL; for these, one of the classes was chosen reflecting expected biological grouping (see Table S4). If a CT is marked as “no mapped parent cell,” the CT term (already crosswalked to CL or PCL) is not a subclass of a top-level CT in the top-n CTs provided by CL. If a CT is marked as “not crosswalked,” this means that the CT label assigned by the CTann tool was not associated with a CL or PCL ID. A full report of cells that are not crosswalked while constructing HRApop but that have been crosswalked semi-manually for Fig. 1 is labeled “unmapped-cell-ids” and is available on GitHub^104^. No general CT could be identified for 617,000 cells that are associated with 10 CTs (called “not mapped parent cell” and shown in gray) and 9 cells are associated with 1 CT in the small and large intestine that are not covered in existing ontologies (called “not crosswalked” and rendered in black).

The DCTA Workflow applies crosswalks after CT annotations are done; CL labels and IDs are used when computing CT populations for datasets, extraction sites, and ASs. Exactly 154 unique cell labels could not be crosswalked to CL and a report of modality, CTann tool (if applicable), organ, and CT label for these in Table S1.

### Existing Code

#### CTann Tools

The three CTann tools in HRApop v1.0 (Azimuth^48^, CellTypist^49,50^, popV^51^) are containerized with Docker in the HRApop CTann Tool Containers on GitHub^62^. The Dockerfile lists operating system requirements, basic setup, and dependencies so the package can be run in the cloud or on a local machine with consistent outputs. This enables the DCTA Workflow to execute them locally. These containers define a dataset handler interface (see code on GitHub^105^) that specifies requirements for every new piece of code that downloads H5AD files from a portal. Docker is used to package each CTann tool along with all of its dependencies into portable containers that can be deployed across different environments. Apptainer (apptainer.org), is used when running HRApop code on HPC clusters. An example for Azimuth is provided on GitHub^106^. Each container has a context file that specifies which models need to be downloaded, such as this one^107^ for Azimuth. Similarly, extracting summaries, computing gene expressions, and crosswalking are also packaged as Docker files. Both Docker and Apptainer/Singularity are supported for running Common Workflow Language (CWL, https://www.commonwl.org) workflows on a Linux cluster for HRApop v1.0. Table S5 lists the name, version number, code base, models used, and requirements for each tool.

#### Mean Biomarker Expressions for sc-transcriptomics Data

Mean biomarker expressions are captured in the DESpop and provided for each CT per dataset. To generate these values, scanpy^108^, numpy^109^, and anndata^110^ are used. Specifically, scanpy’s *rank_gene_groups()* method (scanpy.readthedocs.io/en/stable/generated/scanpy.tl.rank_genes_groups.html) is applied to perform differential expression analysis between CTs. As part of this analysis, this method calculates the mean expression of each gene within a target CT, as well as its expression in the rest of the dataset. These values are used to identify and rank marker genes, and the corresponding mean expressions are recorded for the topn genes, where n is defined by the user. However, it is important to note that this method does not compute mean expression values for all genes across all CTs—only for the most differentially expressed ones. To ensure consistency in gene naming across datasets, gene identifiers are normalized using a lookup table, which maps Ensembl IDs from Release 111^111^ (www.ensembl.org/index.html) to HGNC-approved symbols from version v2023–09-18^112^ (www.genenames.org). This normalization helps maintain interoperability and accuracy when comparing gene expression data across datasets and tools.

#### Tissue Registration

Registering tissue datasets inside the 73 3D reference objects in the HRA is possible via the RUI^24^, which generates 3D extraction sites; it is available as a standalone tool at apps.humanatlas.io/rui but also embedded into the ingest pipelines of HuBMAP and SenNet. The registration process consists of three main phases: assignment, enrichment, and validation.

Initially, the registration coordinator contacts subject matter experts with knowledge of the spatial information associated with tissue samples. Experts can then use the RUI to submit tissue block spatial information themselves or collaborate with the coordinator, who facilitates the submission process with their input. The RUI records spatial information by creating extraction sites inside 3D reference objects. Additionally, it uses a mesh-based collision detection to annotate the extraction with AS tags (see Box 1 and Mesh-Based Collision Detection section). By the end of this phase, all tissue samples should have an assigned extraction site. These workflows are further detailed in SOPs^26–28^.

Once extraction sites have been assigned, enrichment begins. The registration coordinator uses a location processor tool to enhance spatial information with de-identified donor metadata (e.g., sex, age, BMI) and publication metadata (e.g., DOI, authors, publication year). This combined dataset forms a registration set^34^, which is assigned a unique ID for future reference. The code for the processor is accessible via GitHub^113^, as are existing stand-alone HRA Registrations^57^.

Finally, in the validation phase, the expert is asked to review the registration set for accuracy and completeness, requesting revisions if necessary. This process uses a customized instance of the EUI, which the expert uses to evaluate sample locations, metadata accuracy, and AS tags from mesh-based collision detection. Once validated, the registration set is finalized and added to the general EUI (apps.humanatlas.io/eui). This concludes the spatial registration process. An overview of all EUIs for registration sets is available on GitHub^114^.

#### Mesh-Based Collision Detection

To enable more precise collision detection between tissue blocks and ASs, a library for mesh-based collision detection (see Box 1) was created; it is called the HRA Mesh Collision API^115^. Given an extraction site, this HTTP service returns a list of mesh collisions with ASs and metadata. The 3D geometry-based tissue block annotation code includes: (1) a C++ library for the HTTP service for collision detection and intersection volume (see Box 1) computation between extraction sites and ASs, (2) a C++ library for checking manifoldness and closedness of meshes as well as hole-filling for unclosed meshes, and (3) a Python library for converting GLB files to Object File Format (OFF) files, used as the underlying 3D model format for collision detection. Code repository, URL to deployed API, and exemplary API response are available in Table S1.

#### Weighted Cosine Similarity

A collection of functions to compute and use weighted cosine similarities in the RUI2CTpop Workflow is available on GitHub^116^. The script uses math.js (mathjs.org) for access to implementation for the dot product and norm between two vectors (mathjs.org/docs/reference/functions/dot.html and mathjs.org/docs/reference/functions/norm.html).

### New Code

#### HRApop CTann Tool Containers

These are a collection of scripts (see GitHub^62^) for annotating H5AD files using Azimuth^48^, CellTypist^49,50^, and popV^51^. They wrap CTann tools as Docker containers for CWL workflows. This allows each tool to be executed consistently regardless of technology needed, e.g., R vs Python. Default settings are used for each tool where possible. A complete list of settings for each tool is provided in Table S5.

#### DCTA Workflow

HRApop datasets download and CTann is handled by the **DCTA Workflow** (see GitHub^61^). It uses the tools in **HRApop CTann Tool Containers**, to download data from multiple portals after ensuring they are from healthy, human donors, runs applicable CTann tools, analyzes gene expressions to identify top genes, crosswalks CT labels from CTann to ASCT+B tables^35^ using crosswalks, assembles donor metadata, and outputs summarized results for downstream use. It is runnable as a CWL workflow. The CWL runner is written in Python. The DCTA Workflow outputs CT populations and metadata for all annotated sc-transcriptomics and sc-proteomics datasets (see Table S1). They are then copied to the input GitHub repository^117^ for the RUI2CTpop Workflow (see below) for further processing. The Usage Notes section points to example code that runs the DCTA Workflow locally on a user-provided H5AD file.

##### Download

To download H5AD files, the DCTA Workflow constructs a series of jobs to execute. An organ mapping provides crosswalks between organ code names on the portals to Uberon IDs and labels on HuBMAP and SenNet (see this GitHub commit^118^) as well as GTEx (see this GitHub commit^119^). For CxG, no mapping is needed as the metadata contains UBERON IDs already. To retrieve donor metadata across portals, each portal is queried according to their APIs, relevant information is extracted and saved in a harmonized format, see donor field at this GitHub commit^120^. Implementations to extract donor metadata from the different portals are also available, e.g., for SenNet (see this GitHub commit^121^) and CxG (see GitHub^122^).

The DCTA Workflow extracts metadata needed for constructing ds-graphs^22^ (age, sex, BMI, assay type) from the individual portal APIs and saves it as simple JSON files. The H5AD files are downloaded locally into a raw data folder in the DCTA Workflow repository, or onto a file system on a HPC system.

##### Splitting and Re-assembling H5AD Files for GTEx and CxG data

In the data model of HuBMAP and SenNet, donors, organs, tissue blocks, tissue sections, and datasets are modeled as individual entities, where each dataset belongs to exactly one donor. This means that an H5AD file from HuBMAP or SenNet contains data for exactly one donor. On CxG, on the other hand, H5AD files contain multiple donors; to make the two data models work together, H5AD files from CxG need to be split into new H5AD files by donor and organ in the DCTA Workflow. The respective script^123^ is in Python because it needs pandas, a foundational library for data manipulation and analysis (pandas.pydata.org), and anndata for handling annotated data matrices (https://anndata.readthedocs.io/en/stable). This is done to combine donor-organ combinations across assets into new H5AD files. Extracted donor metadata fields are shown in the harmonized donor metadata format described above.

##### Datasets with too few cells

Datasets with fewer than 100 cells are filtered out by the DCTA Workflow. While their H5AD files are downloaded, no CTann is run over them and no CT population is output.

#### RUI2CTpop Workflow

The **RUI2CTpop Workflow** (see GitHub^64^) performs spatial annotation and CT population computation with input files provided by the DCTA Workflow. It sources extraction sites via the HuBMAP and SenNet APIs through HRA API queries at apps.humanatlas.io/api#get-/ds-graph/hubmap, apps.humanatlas.io/api#get-/ds-graph/sennet, and apps.humanatlas.io/api#get-/ds-graph/gtex, with the underlying queries at github.com/x-atlas-consortia/hra-api/blob/main/src/library/ds-graph/operations/hubmap.js, github.com/x-atlas-consortia/hra-api/blob/main/src/library/ds-graph/operations/sennet.js, and github.com/x-atlas-consortia/hra-api/blob/main/src/library/ds-graph/operations/gtex.js. A listing of all extraction sites that are sources is available at https://github.com/x-atlas-consortia/hra-pop/blob/main/input-data/v1.0/config.sh.

For the RUI2CTpop Workflow to function, the DCTA Workflow (see above) provides CT populations and dataset metadata, then those files are copied over to the input folder for a new RUI2CTpop run (see GitHub^84^). Scripts running over these input files during RUI2CTpop are on GitHub^124^. Output data from **RUI2CTpop** is provided on GitHub^125^.

It processes Criteria C1–4 (including a check for donor age to ensure only data from adult humans is used, see Box 1), then gathers extraction sites, CT populations, and donor metadata, including publications. If a dataset has no metadata for age or sex, it is not used for atlas construction. Note that GTEx provides only age ranges, not values, but the data comes from adult donors. It also uses mesh collisions and corridor code to compute corridors (see Corridors section) and to build the ASpop and the DESpop (see both definitions in Box 1, links in Table S1). Exemplary CT populations for a dataset, an extraction site, and an AS are shown at cns-iu.github.io/hra-cell-type-populations-supporting-information/#exemplary-cell-type-populations. Note that in all three cases, the CTann tool(s) are indicated by the annotation_method field. The RUI2CTpop Workflows also contains scripts and SPARQL queries to construct data products for HRApop in the form of CSV reports^58^ to analyze, visualize, validate, and use HRApop data (see link in Table S1).

#### Corridors

For each extraction site with a CT population, a 3D volume of likely origin within the organ is computed, given the biomolecular make-up of the tissue block as represented by its CT population. The result is a complex corridor, i.e., a combined representation for all possible locations, compiled via a shrink-wrap algorithm^126^.

A corridor represents the complete set of spatial positions where an extraction site could plausibly be located, while maintaining its observed intersection ratios with neighboring ASs. Each extraction site is uniquely associated with one such corridor. Corridors are GLB files (see Data Records). In HRApop usage, given a CT population for a dataset of unknown spatial origin, HRApop can be used to predict its spatial origin (HRA US#2). The spatial origin can be an entire AS if it has the same or a similar CT population (measured using weighted cosine similarity) or the extraction site of a tissue block with the most similar CT population (and its corresponding corridor with the same percentages of multiple ASs).

To generate complex 3D corridors given an extraction site, a C++ library with an HTTP service for the 3D Corridor Generation API^127^ given an extraction site made with the RUI was created. A corridor is computed by sending an extraction site to the HRA API at apps.humanatlas.io/api/#post-/v1/corridor (see also Table S1) to get the mesh-based AS. From there, three cases are possible: (1) If the tissue block collides with **only one AS**, the entire AS is returned as a corridor; (2) if the tissue block collides with **two ASs,** a filter-search algorithm is used to efficiently compute all the possible locations before applying the shrink-wrap algorithm to generate a complex corridor. The filter-refine paradigm^128^ is widely used in computationally intensive tasks where infeasible solutions are filtered out from a list of candidates. Next, more viable candidates are examined with respect to their exact geometry to generate exact answers in a refinement step. Inspired by the filter-refine paradigm, a filter-search algorithm is proposed to derive complex corridors. Finally, (3) if a tissue block collides with **three or more ASs,** it is fixed in place, in which case it corresponds exactly to the extraction site.

A listing of all corridors as well as the code, endpoint, and documentation for the 3D Corridor Generation API is available in Table S1. An exemplary corridor belonging to the extraction site with ID 1cbd9283-2d58-4a2d-88fe-effb18c3f14f from the head of the female pancreas that can be inspected in 3D is available on the companion website at cns-iu.github.io/hra-cell-type-populations-supporting-information#exemplary-corridor.

#### Tradeoff between Step Size in Search Stage and Precision of Corridor

Since a sliding window approach is used to search feasible locations, the configuration of the step size is essential. It determines how big a move is made in the search stage (see Table 3). If a large step size is set, locations with the exact intersection volume (see Box 1) with the given extraction site may be skipped. Conversely, if a small step size is set, the computation cost may become overwhelming. Further, the intersection volume from the mesh-based collision detection is returned as a float; thus, if only the exact value is matched, feasible locations can be few. In order to compute corridors with both high precision and reasonable computation overhead, the tolerance for feasible locations can be adjusted to, e.g., 0.1, which means the difference between the intersection volume of the feasible locations and the true intersection volume cannot exceed 10% of the true intersection volume.

## Data Records

Five major HRApop data products are available for download on Zenodo^69^: Atlas ASpop and the DESpop (both as JavaScript Object Notation for Linked Data format, or JSON-LD, see json-ld.org), as well as cell instances (see Box 1) with confidence scores and cell instances with top 10k genes (both as compressed CSV). Additionally, corridors are available as a compressed folder with GLB files.

The Atlas ASpop, DESpop, and corridors are also available via the HRA KG at purl.humanatlas.io/graph/hra-pop/v1.0 and on GitHub^125^, as well as in the form of canned SPARQL queries at apps.humanatlas.io/api/grlc/hra-pop.html. Finally, links to all data are provided in Table S1, as is a complete listing of all code to construct and use HRApop.

Examples for usage of this HRApop Atlas Data are available on the companion website at cns-iu.github.io/hra-cell-type-populations-supporting-information#usage-examples. The Usage Notes section details how to access HRApop data via Jupyter Notebooks.

## Technical Validation

### Heatmaps

While some CTs exist across organs (e.g., macrophages), most CTs are highly specialized to deliver well-defined organ-specific functions in ASs. To demonstrate that ASpop varies not only by organ but also by AS, four heatmaps were made (one per CTann tools plus sc-proteomics, see **Fig. C1** and **Fig. C2** on the companion website at cns-iu.github.io/hra-cell-type-populations-supporting-information#figures. Each heatmap lists CT labels on the x-axis and organ plus AS labels on the y-axis. Table field color represents the scaled mean value (z-score, see Equation 1) for the percentage of CTs identified in each AS. For each heatmap, data from a CTann tool is selected and processed to calculate the average CT percentage associated with all the ASs in an organ. The results are transformed from a data frame into a matrix (CTs by organ plus AS concatenated into a combined label), where each matrix cell represents the average CT percentage measured for CT and AS dyad. Finally, matrix values are converted to a standardized z-score, which is calculated using the formula in Equation 1.

$$Z=\frac{\left(x\;-\;\mu \right)}{\sigma }$$

Equation 1. Computation of standardized z-score for CTs across ASs.

Equation 1 shows x as an average CT percentage measure, μ is the mean average CT percentage, and σ is the standard deviation mean average CT percentage. The z-score identifies how many standard deviations a data point is from the average mean. If the z-score is 0, values are close to the variable’s average; a z-score of 1 indicates that CT percentage values are 1 standard deviation higher than the mean for that CT, values of 2 are 2 standard deviations from the mean, etc.

Differences across organs and CTann become visible. For example, heart, lung, kidney, and pancreas, when annotated with Azimuth (see **Fig. C1A**), show distinct bands of CTs with a z-score of ~1.5, some up to 5. We observe similar patterns for the heart, liver, lung, pancreas, skin, and small and large intestines in CellTypist (see **Fig. C1B**) and breast, heart, liver, lung, pancreas, prostate, skin, small and large intestines, spleen, thymus, urinary bladder, and ureter for popV (see **Fig. C1.C**). High-resolution versions of the heatmaps and the code to generate them are listed on the companion website at cns-iu.github.io/hra-cell-type-populations-supporting-information#z-scores-for-cts-per-organ-and-as.

### Datasets per AS

**Fig. 5.**
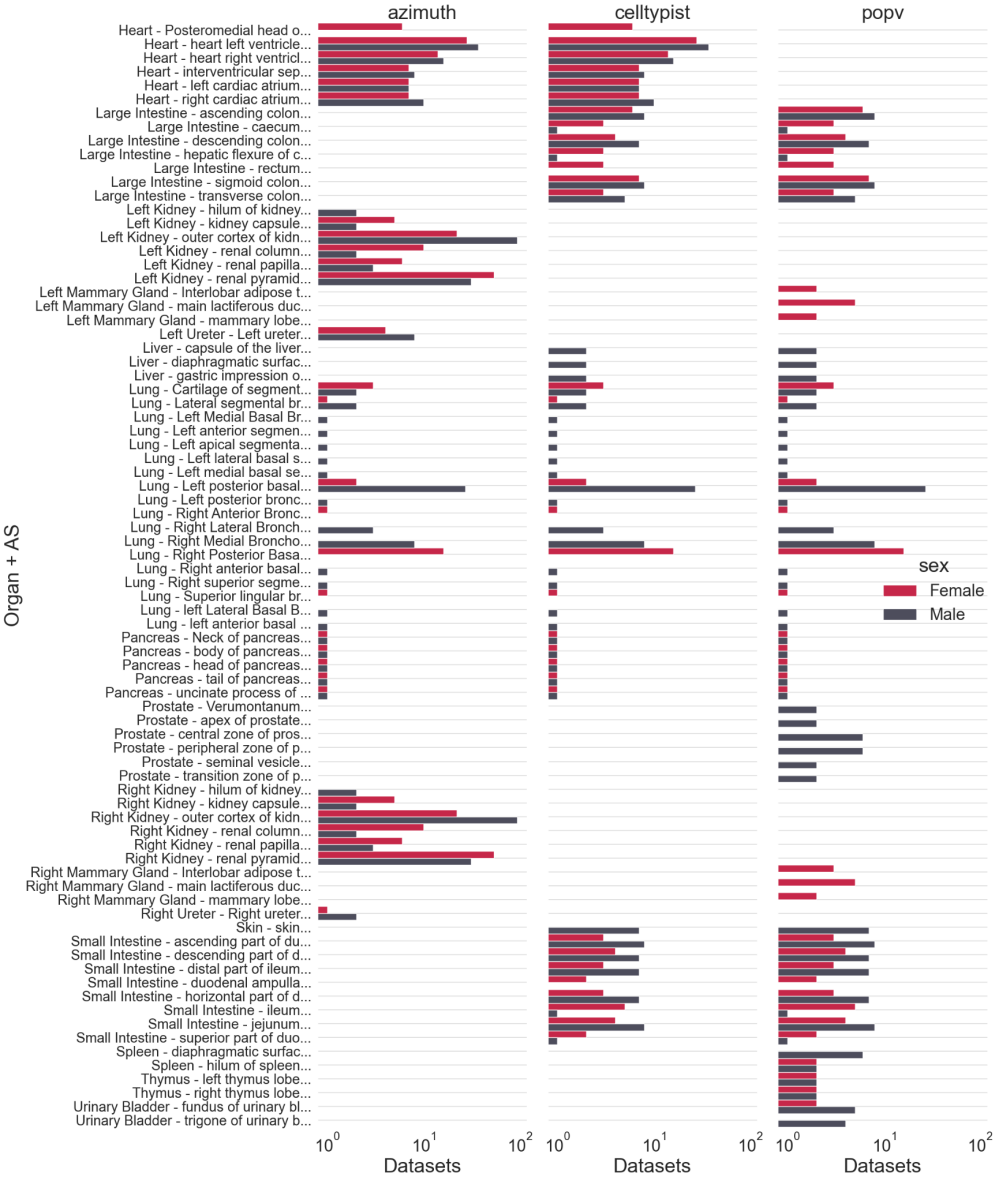
HRApop Atlas Datasets per organ and AS. The number of HRApop Atlas Data per organ and AS combination (y-axis) is plotted on the x-axis, faceted by tool (columns) and colored by sex. Note that the x-axis is scaled logarithmically.

The grouped bar graphs in Fig. 5 show the coverage of HRApop Atlas Data across ASs and organs. Depicted is the number of datasets on the x-axis per AS on the y-axis (labeled by organ for better groupings). Overlaps and gaps in coverage between CTann tools (horizontal), across all organs and ASs (vertical) and between sex (color) become visible. The AS with the most registered HRApop Atlas Data is the outer cortex of the kidney (purl.obolibrary.org/obo/UBERON_0002189), with 75 datasets run through Azimuth. Sc-proteomics datasets are omitted. Counts for this graph are provided on the companion website at cns-iu.github.io/hra-cell-type-populations-supporting-information#dataset-counts.

## Usage Notes

### Annotating local H5AD files with DCTA Workflow

While the DCTA Workflow is designed to run on H5AD files in bulk, code is available to run it on a local H5AD file provided by a user. Details are provided at cns-iu.github.io/hra-cell-type-populations-supporting-information/#running-hra-workflows-runner-on-a-local-h5ad-file. Future development for HRApop will include the ability for the user to upload H5AD files to a web server and get a CT population in return.

### Getting HRApop Data via API Queries

A Jupyter Notebook detailing easy access to CT populations for ASs, extraction sites, and datasets is available at cns-iu.github.io/hra-cell-type-populations-supporting-information#accessing-hrapop-data.

### Visualizing CT Populations for ASs, Extraction Sites, and Datasets

A web interface to create bar graph visualizations for HRApop Atlas CT populations is described and available at cns-iu.github.io/hra-cell-type-populations-supporting-information/#visualizing-cell-type-populations-for-as-es-and-datasets.

### No-code Access to HRApop Data and Functionality

No-code UIs were implemented to allow users to enter a 3D extraction site and get a predicted CT population (Cell Population Predictor, HRA US#1) or enter a CT population and get a predicted spatial origin (Tissue Origin Predictor, HRA US#2). These predictions made with HRApop are at the AS, not the organ level. Details for both UIs are provided below.

#### Cell Population Predictor (US#1)

The demo application at apps.humanatlas.io/us1 uses the ASpop to predict a CT population for a user-provided extraction site with unknown CTs and numbers. A screenshot is shown in Fig. S3.

The user can either (a) create an extraction site with the RUI, (b) upload an existing one, or (c) use a sample extraction site^129^ from the male, left kidney. After clicking the Generate Summary button, the user is presented with a CT population based on the extraction site. Behind the scenes, the application sends the extraction site to the HRA Mesh Collision API, gets the AS tags and intersection percentages (see Box 1), then calculates the resulting CT population based on the ASpop for any colliding AS for which HRApop data exists. As a result, the user gets a table with one row per CT, and with columns for the CTann tool, the modality, the percentage of that CT for the total summary, a raw count, a cell label, and a cell ID. The API call used by the demo is documented at apps.humanatlas.io/api/#post-/hra-pop/rui-location-cell-summary. The code for this application is available on GitHub^130^. HRA US#1 can be used to (1) predict CT populations for datasets for which no assays have been performed yet or (2) perform QA/QC on an assay by validating its results against a query in HRApop. The code and API calls used by this demo are documented in Table S2.

#### Tissue Origin Predictor (US#2)

The demo application at apps.humanatlas.io/us2 uses the ASpop and the DESpop to predict the most similar ASs for which HRApop data exist, the most similar extraction sites, and the most similar HRApop datasets for a user-provided CT population of a dataset with unknown spatial origin. A screenshot is shown in Fig. S4.

The user can either (a) upload a CSV file with a CT population for a dataset or (b) use a sample CSV file^131^ with a CT population for the heart. Then, the user is asked to choose the organ of origin from a dropdown list, as well as the CTann tool with which the CT population was created (Azimuth, CellTypist, or popV). When clicking the Generate Predictions button, three types of possible spatial origin are presented, all sorted by weighted cosine similarity (see Methods section) between the AS and the user-provided CT population: (1) a list of similar ASs, (2) a list of similar datasets; and (3) a link to an EUI with the most similar extraction sites. To retrieve these, the weighted cosine similarity between the vector representing the user-provided CT population and the HRApop ASpop is computed. This is a value between 0 (no shared CT counts) and 1 (same CT counts). The API call used by the demo is documented at apps.humanatlas.io/api/#post-/hra-pop/rui-location-cell-summary. The code for this application is available on GitHub^132^. The code and API calls used by this demo are documented in Table S2.

## Supplementary Material

Supplement 1

## Figures and Tables

**Table 1.** HRApop Atlas Datasets. A breakdown of datasets that meet Criteria C1-4 (see Box 1) and are used to construct HRApop v1.0.

Sex	Consortium	#Datasets	#Cells	Modality
Female	GTEx	7	47,863	sc_transcriptomics
Male	GTEx	8	70,113	sc_transcriptomics
Female	HCA	63	364,993	sc_transcriptomics
Male	HCA	58	359,273	sc_transcriptomics
Female	HuBMAP	22	900,547	sc_proteomics
Female	HuBMAP	112	2,933,015	sc_transcriptomics
Male	HuBMAP	82	15,676,316	sc_proteomics
Male	HuBMAP	253	6,626,620	sc_transcriptomics
Female	NHLBI/LungMap	1	4,680	sc_transcriptomics
Male	NHLBI/LungMap	2	5,713	sc_transcriptomics
Female	SenNet	18	225,940	sc_transcriptomics
Male	SenNet	36	404,540	sc_transcriptomics
**TOTAL**		**662**	**27,619,613** sc-transcriptomics: **11,042,750** sc-proteomics: **16,576,863**	

**Table 2.** Number of datasets, extraction sites, ASs, and organs covered in HRApop v1.0.

	Datasets	RUI extraction sites	ASs covered	Organs covered
**Input data for RUI2CTpop Workflow**	16,293	1,132	164	49
**HRApop Atlas**	662	230	73	17

**Table 3.** Filter and search stage for pre-computing corridors.

1	**Filter stage**	Each collided mesh is approximated by their minimum bounding boxes. Then, the area ***Ω*** where a fixed-size, axis-aligned tissue block can be put is computed so that it can intersect with all the minimum bounding boxes of meshes.
2	**Search stage**	A brute force search algorithm is applied by specifying the step size. A sliding window approach is used to move the fixed-size tissue block within the searching area ***Ω*** computed in the filter stage.

## Box 1.

Key terminology used to explain HRApop. Terms in *italics* have their own entry, either in this Box or in the HRA marker paper^34^ or the HRA KG paper^22^. The former already contains definitions for: *3D collision, extraction site, AS, ASCT+B table, biomarker, CT, CTann tool, crosswalk, millitome, polygon mesh, registration set, RUI registered, sc-transcriptomics/sc-proteomics data, tissue block,* and *(weighted) cosine similarity*. The latter contains definitions for: *3D reference object, ds-graph*, and *SPARQL* (https://www.w3.org/TR/sparql11-query).

- **AS Cell Type Population (ASpop)**: Captures the number of cells per CT for an *AS* given with an ontology ID, donor sex, *AS* label, the *CTann tool* used to assign *CTs*, and a list of datasets from which the data was sourced. An exemplary *ASpop* is shown on the companion website at cns-iu.github.io/hra-cell-type-populations-supporting-information/#for-an-as.
- **Cell instance**: Is an occurrence of a unique cell, identified by its bar code or a similar unique ID. In the data products of HRApop v1.0 (see Data Records), cells can be annotated by multiple CTann, which leads to potentially multiple occurrences of the same cell.
- **Cell type population**: Is a listing of unique CTs and their counts computed for *ASs, extraction sites*, and datasets. For the latter, mean B expression values are also computed. *CT populations* are computed from *CT* counts in experimental datasets, obtained either via *CTann* in the *DCTA Workflow* (for *sc-transcriptomics* datasets), or via expert/author-provided annotations (*sc-proteomics* datasets). *CT populations* are associated with exactly one *CTann* tool; if a dataset has *CT populations* from multiple *CTann* tools, the *CTann* tool that computed the CT population becomes part of its provenance. *CT populations* are kept inside the *ASpop* graph (for an entire AS) and the *DESpop* (for datasets and extraction sites).
- **Collision detection (bounding box)**: Is a computationally efficient but imprecise method for identifying intersections between two 3D objects in Euclidean space. For each 3D object, the bounding box is a cuboid shape that is calculated that encapsulates all vertices and only this bounding box is used when computing collisions.
- **Collision detection (mesh-based)**: A more precise but computationally more expensive method for identifying the intersection between two 3D objects in Euclidean space based on mesh surfaces, resulting in *AS* tags for extraction sites. To enable mesh-based collision detection, *3D reference objects* need to be pre-processed. Details are provided in the Methods section.
- **Corridor**: Describes a 3D volume that encompasses all possible locations for an *extraction site* while maintaining its *intersection percentages* with any *ASs* based on *mesh collision detection*.
- **Criteria C1–4**: Describes four conditions that a dataset must fulfill in order to be used for HRApop construction: (1) it has an extraction site in a 3D reference object, (2) it has a *CT population* via *CTann* or the *sc-proteomics* workflow, (3) it comes from a data portal with QA/QC or have an associated peer-reviewed publication in a scientific journal, and (4) it is from a healthy adult human donor.
- **Dataset and Extraction Site Cell Type Populations (DESpop, formerly “Atlas-Enriched Dataset Graph” in a related HRA publication**^34^): Is a collection of datasets that meet *Criteria C1–4* and are used for HRApop construction. A snippet of the *DESpop* is shown on the companion website at cns-iu.github.io/hra-cell-type-populations-supporting-information/#for-a-dataset.
- **D****ownload and** **C****ell** **T****ype** **A****nnotation (DCTA) Workflow**: Is a set of scripts to download H5AD files, execute the Docker (www.docker.com) containers in *HRApop CTann Tool Containers*, and output CT populations and data metadata as input for the RUI2CTpop Workflow.
- **Donor**: Is an individual person, living or deceased, who contributed tissue. Donors have demographic metadata, such as age, body mass index (BMI), race/ethnicity, and sex. All *donors* in HRApop are healthy, human adults.
- **Experimental dataset**: Describes cell by gene matrices (via *CTann tools*) or cell by protein matrices via Organ Mapping Antibody Panels (OMAPs)^56^ derived from an organ-specific *tissue block* from a *donor*.
- **HRApop Atlas Data (also called HRApop Data Used in Atlas Construction)**: Is a collection of high-quality experimental datasets that fulfill *Criteria C1–4* that is used in atlas construction. Also part of the *HRApop Atlas* are the *CT populations of* 3D extraction sites of these datasets, plus the ASs with which these extraction sites collide. At the beginning is a programmatically compiled collection of dataset IDs, extraction sites, and donor metadata that forms the input of the *DCTA Workflow*. Dataset IDs can come from three sources: (1) sc-portals with access to sc-transcriptomics H5AD files (from HuBMAP, SenNet, CxG, and GTEx), which are then given to the *DCTA Workflow* as an input, (2) sc-proteomics datasets, and (3) HRA Registrations, see GitHub^57^ and Table S1, a set of manually curated extraction sites, from which the *RUI2CTpop Workflow* extracts dataset IDs and attempts to match them with *CT populations* from the *DCTA Workflow*. Each HRApop run provides a series of reports (see GitHub^58^).
- **HRApop CTann Tool Containers**: Is a collection of CTann tools containerized with Docker to annotate H5AD files. The DCTA Workflow uses the *HRApop CTann Tool Containers*. They also contain utilities for CTann, e.g., computing gene expressions and identifying the top-n genes.
- **HRApop Prediction Data**: Datasets that fulfill C3–4 *and* have either *CT populations* (fulfilling C2) or *extraction sites* (fulfilling C1) but not both and for which the missing information can be predicted.
- **Intersection percentage or volume**: Is a measure that describes the shared 3D space between two 3D objects (e.g., a *tissue block* and an *AS*) expressed as a percentage of the total volume or as an absolute value in cubic millimeters (mm^3^).
- **Mean biomarker expressions**: Are computed for the top 10 genes per CT per dataset, then stored in the *DESpop*.
- **RUI** **to Compile** **CTpop** **(RUI2CTpop) Workflow**: Is a collection of scripts to compile the *HRApop Atlas Data* from the output of the *DCTA Workflow* (see Fig. 2 and Methods).
