# Supplementary material for: Constructing and Using Cell Type Populations of the Human Reference Atlas
Source: bioRxiv. 2025 Aug 20:2025.08.14.670406. Preprint. [Version 1] doi: 10.1101/2025.08.14.670406 (PMC12393406; doi:10.1101/2025.08.14.670406)
Supplement: Supplement 1 [file NIHPP2025.08.14.670406v1-supplement-1.pdf]

bioRxiv preprint doi: https://doi.org/10.1101/2025.08.14.670406; this version posted August 20, 2025. The copyright holder for this preprint (which was not certified by peer review) is the author/funder, who has granted bioRxiv a license to display the preprint in perpetuity. It is made available under aCC-BY 4.0 International license.

# Supplemental Figures

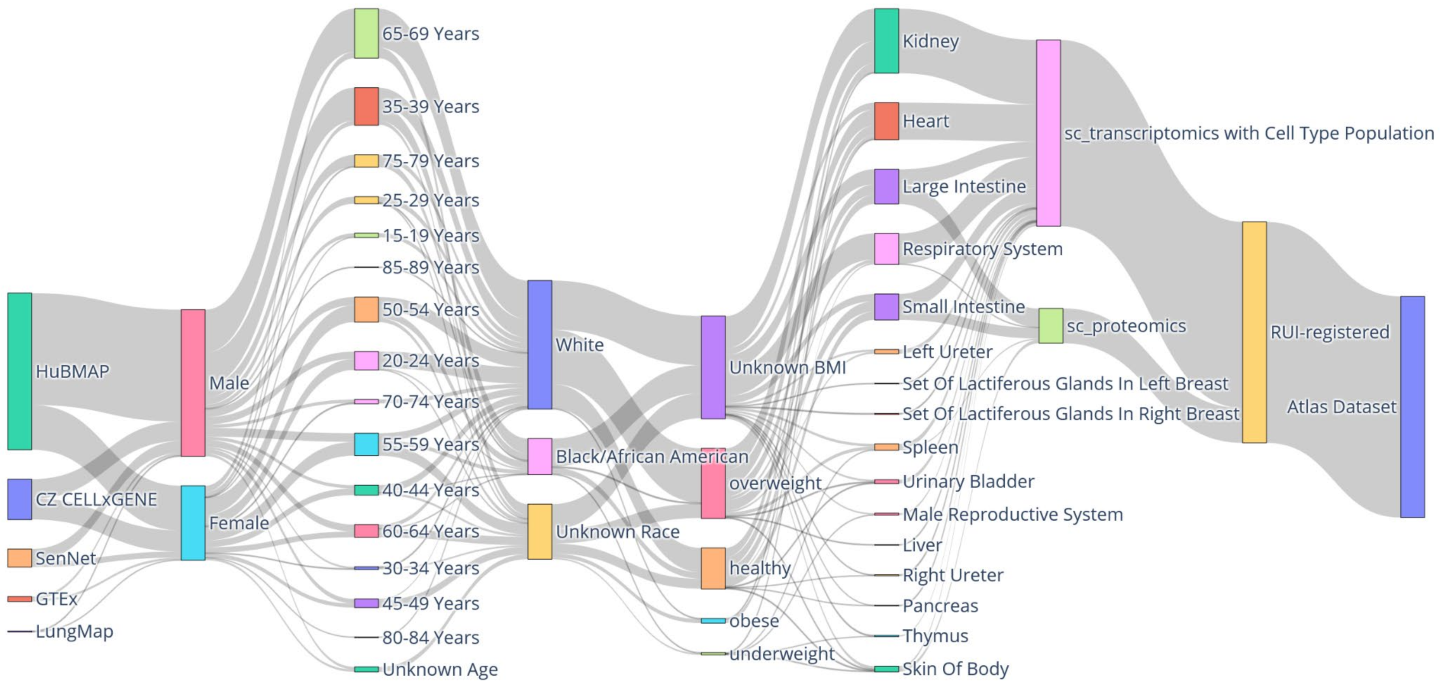


**Fig. S1. A Sankey Diagram for HRApop Atlas Data.** Interactive data visualization can be explored at [cns-iu.github.io/hra-cell-type-populations-supporting-information/sankey_atlas_plotly.html](cns-iu.github.io/hra-cell-type-populations-supporting-information/sankey_atlas_plotly.html).

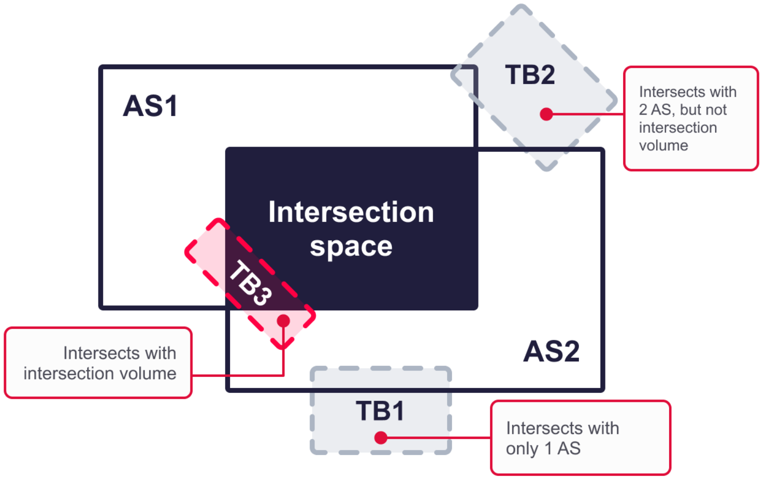


**Fig. S2. Intersection space and intersection volumes.** Two intersecting ASs create a 3D intersection space. Three exemplary tissue blocks (TB) intersect with one AS (TB1), both ASs (TB2), or both and their intersection space (TB3).

bioRxiv preprint doi: https://doi.org/10.1101/2025.08.14.670406; this version posted August 20, 2025. The copyright holder for this preprint (which was not certified by peer review) is the author/funder, who has granted bioRxiv a license to display the preprint in perpetuity. It is made available under aCC-BY 4.0 International license.

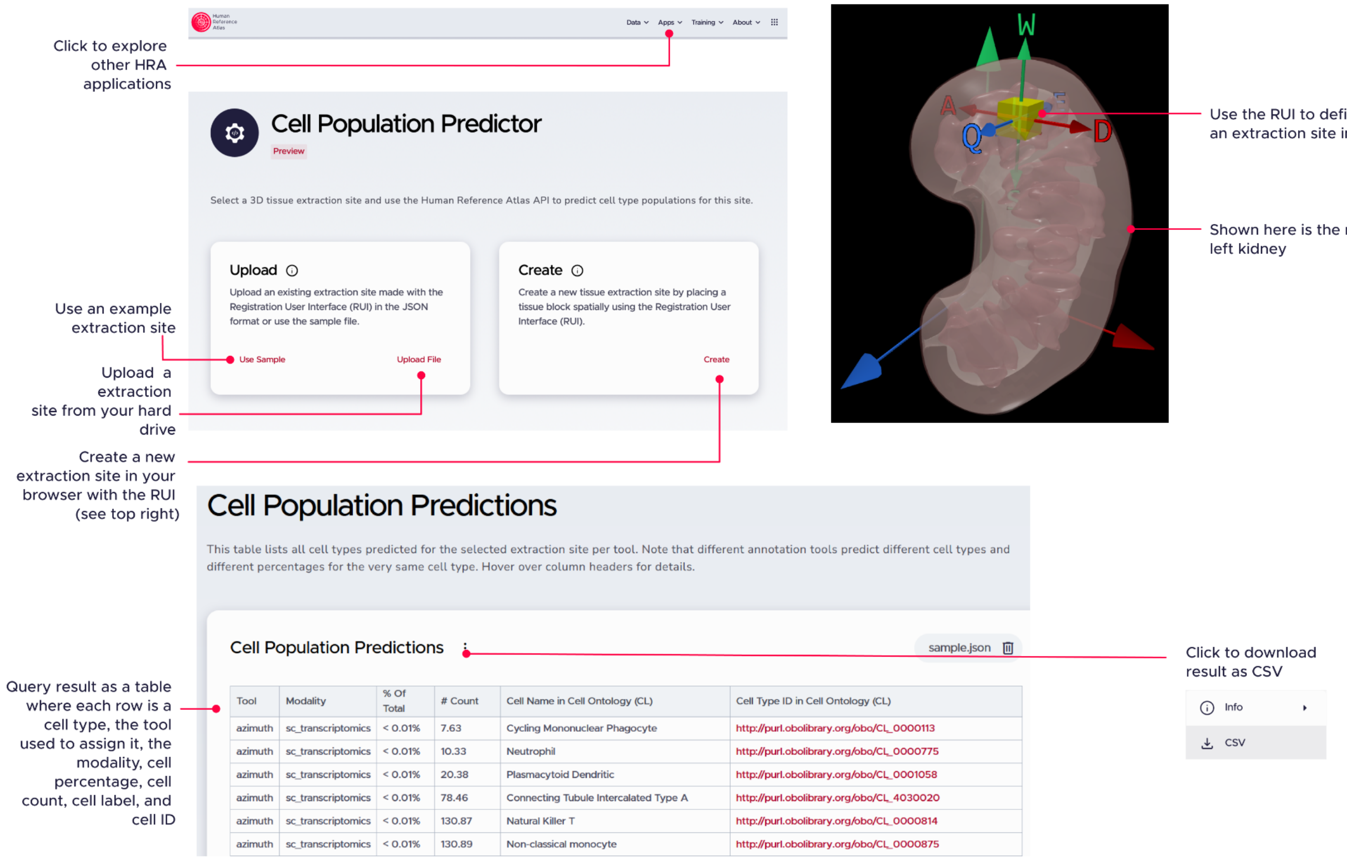


**Fig. S3. Annotated screenshot of the Cell Population Predictor.**

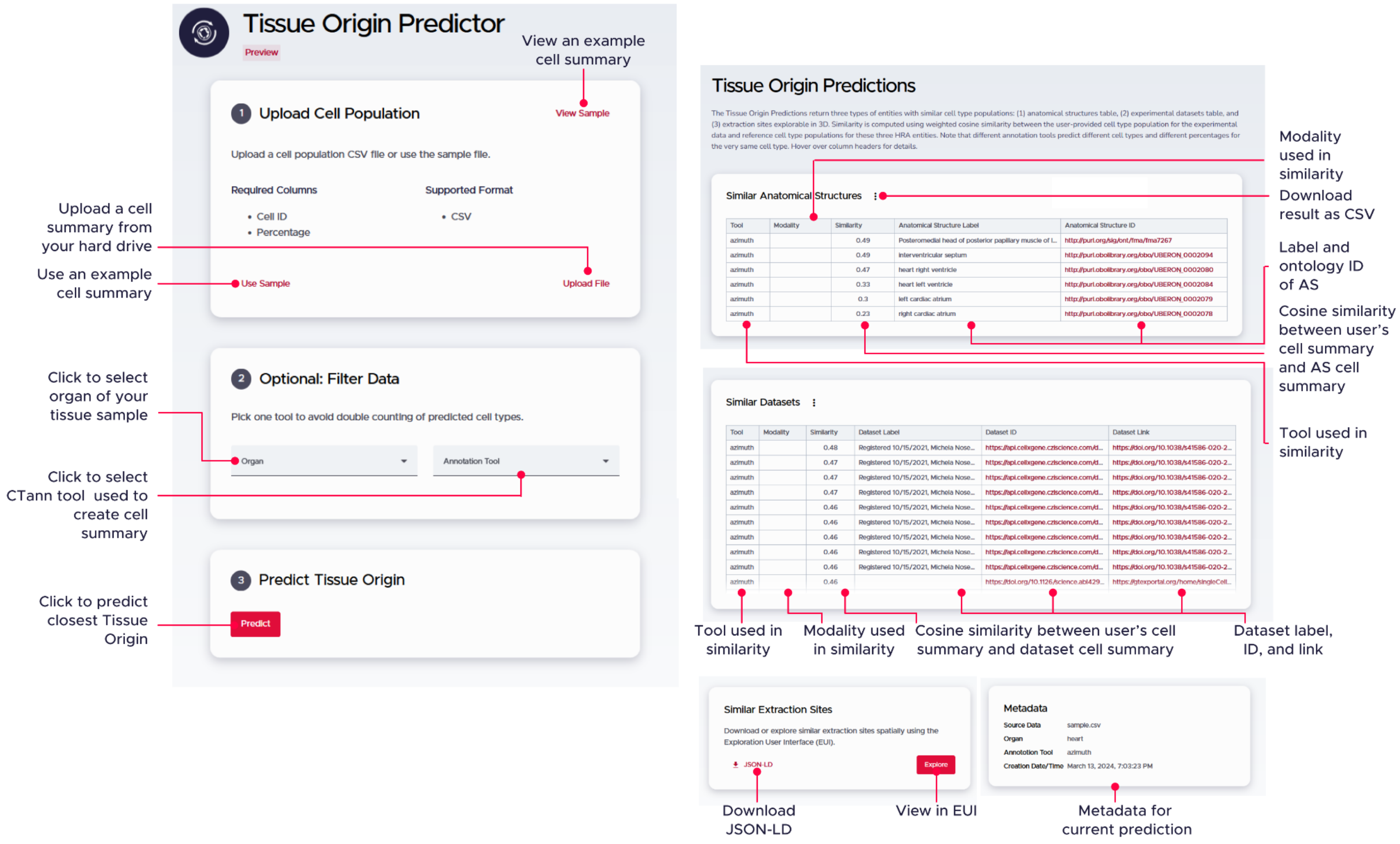


**Fig. S4. Annotated screenshot of the Tissue Origin Predictor.**

bioRxiv preprint doi: https://doi.org/10.1101/2025.08.14.670406; this version posted August 20, 2025. The copyright holder for this preprint (which was not certified by peer review) is the author/funder, who has granted bioRxiv a license to display the preprint in perpetuity. It is made available under aCC-BY 4.0 International license.

# Supplemental Tables

**Table S1. Listing of all GitHub repositories used to construct and use HRApop**. Note that the HRA US#1 and US#2 Demo Applications are supported by the HRA KG, the HRA Digital Object Processor, and the HRA API. Please see details in the HRA KG paper[22].

| Name | Description | URL |
|---|---|---|
| **Data** | | |
| HRApop Atlas | ASpop and DESpop | Zenodo[69]<br><br>HRA KG: purl.humanatlas.io/graph/hra-pop/v1.0 |
| DESpop | Provided in a variety of formats (including JSON-LD and Terse RDF Triple Language [Turtle][135]) via the HRA KG and as JSON-LD on GitHub | Zenodo[69]<br><br>GitHub (JSON-LD): github.com/x-atlas-consortia/hra-pop/blob/main/output-data/v1.0/atlas-enriched-dataset-graph.jsonld<br><br>For datasets on GitHub (CSV): github.com/x-atlas-consortia/hra-pop/blob/main/output-data/v1.0/reports/atlas-ad-hoc/cell-types-per-dataset.csv<br><br>For datasets on grlc.io (canned SPARQL query): apps.humanatlas.io/api/grlc/hra-pop.html#get-/cell-types-per-dataset<br><br>For extraction sites on GitHub (CSV): github.com/x-atlas-consortia/hra-pop/blob/main/output-data/v1.0/reports/atlas-ad-hoc/cell-types-per-extraction-site.csv<br><br>For extraction sites on grlc.io (canned SPARQL query): apps.humanatlas.io/api/grlc/hra-pop.html#get-/cell-types-per-extraction-site<br><br>HRA KG: cdn.humanatlas.io/digital-objects/graph/hra-pop/latest/assets/atlas-enriched-dataset-graph.jsonld |
| ASpop | Provided via the HRA KG and via GitHub | Zenodo[69]<br><br>HRA KG: cdn.humanatlas.io/digital-objects/graph/hra-pop/v1.0/assets/atlas-as-cell-summaries.jsonld<br><br>GitHub (JSON-LD): github.com/x-atlas-consortia/hra-pop/blob/main/output-data/v1.0/atlas-as-cell-summaries.jsonld<br><br>GitHub (CSV): github.com/x-atlas-consortia/hra-pop/blob/main/output-data/v1.0/reports/atlas-ad-hoc/cell-types-in-anatomical-structurescts-per-as.csv<br><br>grlc.io (canned SPARQL query): apps.humanatlas.io/api/grlc/hra-pop.html#get-/cell_types_in_anatomical_structurescts_per_as |

bioRxiv preprint doi: https://doi.org/10.1101/2025.08.14.670406; this version posted August 20, 2025. The copyright holder for this preprint (which was not certified by peer review) is the author/funder, who has granted bioRxiv a license to display the preprint in perpetuity. It is made available under aCC-BY 4.0 International license.

| | | |
|---|---|---|
| Cell instances | Cell instances for sc-transcriptomics datasets in HRApop v1.0 | Zenodo[69] |
| Cell instances with top-10,000 biomarkers | Cell instances for sc-transcriptomics datasets in HRApop v1.0 with top-10,000 biomarkers | Zenodo[69] |
| Corridors | Corridors for extraction sites for HRApop v1.0 | Zenodo[69]<br><br>GitHub:<br>github.com/x-atlas-consortia/hra-pop/blob/main/output-data/v1.0/atlas-as-cell-summaries.jsonld<br><br>HRA KG:<br>lod.humanatlas.io/graph/hra-pop/v1.0/ |
| HRApop Prediction Data | As of HRApop v1.0, there are 617 unique extraction sites for which a CT population can be predicted (US#1) and 5,453 unique datasets for which a spatial original can be predicted (US#2) | US#1: github.com/x-atlas-consortia/hra-pop/blob/main/output-data/v1.0/reports/atlas/application-a1.csv<br><br>US#2: github.com/x-atlas-consortia/hra-pop/blob/main/output-data/v1.0/reports/atlas/application-a2p1.csv |
| **Code (Construction)** | | |
| HRApop CTann Tool Containers | Has Docker containers for running CTann tools over H5AD files. | Repository: GitHub[62]<br><br>Containers: GitHub[136] |
| DCTA Workflow | A set of scripts to download H5AD files, execute the Docker containers in HRApop CTann Tool Containers, and output CT populations and data metadata as input for the RUI2CTpop Workflow. | GitHub[61] |
| CTann crosswalks | A collection of CSV files that link CT labels from Azimuth, CellTypist, and popV to CL or PCL IDs so they can be connected to the ASCT+B tables and other HRA Digital Objects. For example, the most recent crosswalk CSV file for Azimuth is available for download at the bottom of | lod.humanatlas.io/ctann |

bioRxiv preprint doi: https://doi.org/10.1101/2025.08.14.670406; this version posted August 20, 2025. The copyright holder for this preprint (which was not certified by peer review) is the author/funder, who has granted bioRxiv a license to display the preprint in perpetuity. It is made available under aCC-BY 4.0 International license.

| | | |
|---|---|---|
| | the HRA Digital Object landing page at lod.humanatlas.io/ctann/azimuth/latest. | |
| CTs level mapping | Report and queries to map crosswalked, unique CTs to higher-level CTs | Report:<br>github.com/x-atlas-consortia/hra-pop/blob/main/output-data/v1.0/reports/atlas-ad-hoc/cell-types-level-mapping.csv<br><br>Query:<br>github.com/x-atlas-consortia/hra-pop/blob/main/queries/atlas-ad-hoc/cell-types-level-mapping.rq<br><br>Report (long):<br>github.com/x-atlas-consortia/hra-pop/blob/main/output-data/v1.0/reports/atlas-ad-hoc/cell-types-level-mapping-long.csv<br><br>Query:<br>github.com/x-atlas-consortia/hra-pop/blob/main/queries/atlas-ad-hoc/cell-types-level-mapping-long.rq |
| Not crosswalked | Reports for cell IDs that were not crosswalked during the DCTA Workflow | Input for RUI2CTpop Workflow: GitHub[137]<br><br>HRApop Atlas: GitHub[104] |
| CT populations and metadata for sc-transcriptomics and sc-proteomics data, produced by the DCTA Workflow and serving as input for the RUI2CTpop Workflow | **sc-transcriptomics-cell-summaries.jsonld.gz**: Contains CT populations for all sc-transcriptomics datasets annotated with a CTann tool in JSON-LD.<br><br>**sc-transcriptomics-dataset-metadata.csv**: Contains organ, donor metadata, . It also features a handler ID to denote which portal a dataset was downloaded from, a UUID, an assay type, and tissue provider information.<br><br>**sc-proteomics-cell-summaries.jsonld:** Contains CT populations for all sc-proteomics datasets in JSON-LD. The summaries are produced via the DCTA Workflow using the code at github.com/cns-iu/hra-node-dist-vis/blob/main/scripts/build- | GitHub (all)[117]<br><br>GitHub (cell summaries for sc-protemics only)[84] |

bioRxiv preprint doi: https://doi.org/10.1101/2025.08.14.670406; this version posted August 20, 2025. The copyright holder for this preprint (which was not certified by peer review) is the author/funder, who has granted bioRxiv a license to display the preprint in perpetuity. It is made available under aCC-BY 4.0 International license.

| | | |
|---|---|---|
| | cell-summaries.js, which queries a listing of all possible sc-proteomics datasets (see GitHub[83]).<br><br>**sc-transcriptomics-dataset-metadata.csv:** Contains donor, tissue block, and data IDs for sc-proteomics datasets. | |
| RUI2CTpop Workflow | A collection of scripts to compile HRApop from the output of the DCTA Workflow, which first provides CT populations and dataset metadata, then copies those files over to GitHub[117]. Scripts running over these input files are also on GitHub[124]. Output data is provided at github.com/x-atlas-consortia/hra-pop/tree/main/output-data/v1.0. For downstream analysis, reports based on SPARQL queries are on GitHub[58].<br><br>A full log is available at raw.githubusercontent.com/x-atlas-consortia/hra-pop/refs/heads/main/output-data/v1.0/log.txt.<br><br>A file to capture the creation date of the pipeline having finished is at github.com/x-atlas-consortia/hra-pop/blob/main/output-data/v1.0/CREATION_DATE.<br><br>A Readme for the most recent run is present at github.com/x-atlas-consortia/hra-pop/blob/main/output-data/v1.0/README.md. | GitHub[64] |
| **Code (Support)** | | |

bioRxiv preprint doi: https://doi.org/10.1101/2025.08.14.670406; this version posted August 20, 2025. The copyright holder for this preprint (which was not certified by peer review) is the author/funder, who has granted bioRxiv a license to display the preprint in perpetuity. It is made available under aCC-BY 4.0 International license.

| | | |
|---|---|---|
| HRA Registrations | Manually curated HRA Dataset Graphs. This repository holds static dataset graphs for use in the EUI and other HRA applications. All registration Digital Objects are published to hubmapconsortium.github.io/hra-registrations/. All registrations are at hubmapconsortium.github.io/hra-registrations/**name-in-root-folder**/rui_locations.jsonld | GitHub[57] |
| HRA Registrations Processor | Command line interface to simplify creating rui_locations.jsonld files | github.com/hubmapconsortium/hra-rui-locations-processor |
| Querying HRApop | A collection of SPARQL queries for use in this paper and for downstream analysis (to identify counts and get CT populations, biomarkers) | grlc.io/api-git/hubmapconsortium/ccf-grlc/subdir/hra-pop/ |
| RUI | Stand-alone RUI use to register tissue blocks | Stand-alone RUI: apps.humanatlas.io/rui/<br><br>RUI code repository: github.com/hubmapconsortium/hra-ui/tree/main/apps/ccf-rui |
| EUI | Deployed stand-alone RUI extraction site sets displayed in the EUI | hubmapconsortium.github.io/hra-registrations |
| CTann tools | Azimuth: v0.4.6<br>CellTypist: v1.6<br>popV: github.com/YosefLab/popV/tree/2d29c9a290d2015ec65ef0ef9f0e6b6d2277e7bb | Azimuth: azimuth.hubmapconsortium.org<br>CellTypist: www.celltypist.org<br>popV: github.com/YosefLab/PopV |
| **Collision Detection and Corridors** | | |
| HRA Mesh Collision API | Given an extraction site, get mesh-based collisions with the 3D reference object. | Code: github.com/hubmapconsortium/hra-tissue-block-annotation<br><br>Deployed: pfn8zf2gtu.us-east-2.awsapprunner.com/get-collisions<br><br>Public endpoint: apps.humanatlas.io/api/v1/collisions<br><br>Documentation for endpoint: apps.humanatlas.io/api/#post-/v1/collisions<br><br>Example result: |

bioRxiv preprint doi: https://doi.org/10.1101/2025.08.14.670406; this version posted August 20, 2025. The copyright holder for this preprint (which was not certified by peer review) is the author/funder, who has granted bioRxiv a license to display the preprint in perpetuity. It is made available under aCC-BY 4.0 International license.

| | | |
|---|---|---|
| | | github.com/hubmapconsortium/hra-tissue-block-annotation/blob/main/examples/test-registration-collisions.json |
| 3D Corridor Generation API | Given an extraction site, generate a corridor with the 3D reference object as a GLB file | Code: github.com/hubmapconsortium/hra-corridor-generation<br><br>Deployed: dwwcpwad72.us-east-2.awsapprunner.com/get-corridor<br><br>Public endpoint: apps.humanatlas.io/api/v1/corridor<br><br>Documentation for endpoint: apps.humanatlas.io/api/#post-/v1/corridor |
| Corridors for downloading | Contains all corridor GLB files | github.com/x-atlas-consortia/hra-pop/tree/main/output-data/v1.0/corridors |
| Mesh-mesh collision and annotation | | Code: github.com/hubmapconsortium/hra-glb-mesh-collisions<br><br>On PyPi: pypi.org/project/hra-glb-mesh-collisions/0.1.0/ |
| **Coverage and Visualization** | | |
| CT Populations by AS | Provides a query for an overview of all AS-CT combinations in ASpop of HRApop v1.0, with sex, tool, CT, and cell percentage | apps.humanatlas.io/api/grlc/hra-pop.html#get-/cell_types_in_anatomical_structurescts_per_as. |
| Supporting Information for this paper | Contains assets for the companion website at cns-iu.github.io/hra-cell-type-populations-supporting-information/ | github.com/cns-iu/hra-cell-type-populations-supporting-information |
| Sankey Diagram for HRApop Atlas | Contains the deployed Sankey diagram for HRApop Atlas | cns-iu.github.io/hra-cell-type-populations-supporting-information/sankey_atlas_plotly.html |
| Sankey Diagram for input for RUI2CTpop Workflow | Contains the deployed Sankey diagram for the input for RUI2CTpop Workflow | cns-iu.github.io/hra-cell-type-populations-supporting-information/sankey_universe_plotly.html |
| HRApop Counts | Contains numbers reported in this paper | github.com/cns-iu/hra-cell-type-populations-supporting-information/blob/main/counts/hra_pop_counts.ipynb |

bioRxiv preprint doi: https://doi.org/10.1101/2025.08.14.670406; this version posted August 20, 2025. The copyright holder for this preprint (which was not certified by peer review) is the author/funder, who has granted bioRxiv a license to display the preprint in perpetuity. It is made available under aCC-BY 4.0 International license.

**Table S2. Listing of all HRA applications that use HRApop data.**

| Name | Description | URL[17] |
|---|---|---|
| FTU Explorer | Web-deployed UI to view and explore cell type populations from experimental datasets for 2D FTU illustrations | apps.humanatlas.io/ftu-explorer/#/ |
| HRA Organ Gallery[65,138] | Enables an immersive view of the HRA by showing 71 reference organs and 1,100+ tissue blocks alongside CT populations in VR[65,138]. | humanatlas.io/hra-organ-gallery |
| HRA API | Provides programmatic access to the HRA, enabling integration with other applications and services. A SPARQL endpoint for the HRA API allows users to write their own SPARQL queries. Canned queries for HRApop are available at apps.humanatlas.io/api/grlc/hra-pop.html. | API documentation: apps.humanatlas.io/api/<br><br>The HRA API on the HRA Portal: humanatlas.io/api |
| Cell Population Predictor | A web application where the user can (1) generate a new extraction site, (2) upload an existing extraction site in JSON-LD, or (3) use a pre-made extraction site, then receive a CT population based on HRApop data given the AS mesh collisions of the extraction site. | Deployed at: apps.humanatlas.io/us1/<br><br>On HRA Portal: humanatlas.io/user-story/1<br><br>Code: github.com/x-atlas-consortia/hra-apps/tree/main/applications/us1-spatial-to-cell<br><br>HRA API endpoints used: apps.humanatlas.io/api/hra-pop/rui-location-cell-summary |
| Tissue Origin Predictor | A web application where the user can (1) upload their own CT population or (2) use a provided one, indicate organ and CTann tool used, and then receive (a) a list of similar ASs with CT populations (weighted cosine similarity, see **Methods** section), (b) a list of most similar datasets (weighted cosine similarity), and (c) an EUI with the most similar extraction sites. | Deployed at: apps.humanatlas.io/us2/<br><br>On HRA Portal: humanatlas.io/user-story/2<br><br>Code: github.com/x-atlas-consortia/hra-apps/tree/main/applications/us2-cell-to-spatial<br><br>HRA API endpoints used: apps.humanatlas.io/api/#post-/hra-pop/cell-summary-report<br>apps.humanatlas.io/api/#get-/hra-pop/supported-organs<br>apps.humanatlas.io/api/#get-/hra-pop/supported-tools |

bioRxiv preprint doi: https://doi.org/10.1101/2025.08.14.670406; this version posted August 20, 2025. The copyright holder for this preprint (which was not certified by peer review) is the author/funder, who has granted bioRxiv a license to display the preprint in perpetuity. It is made available under aCC-BY 4.0 International license.

**Table S3. Reports about dataset and cell counts from the RUI2CTpop Workflow.**

| **Counts** | | |
|---|---|---|
| Input for RUI2CTpop Workflow: sc-transcriptomics cell counts | Contains the number of annotated cells in sc-transcriptomics datasets that are part of the input for RUI2CTpop Workflow | github.com/x-atlas-consortia/hra-pop/blob/main/output-data/v1.0/reports/universe-ad-hoc/universe-sc-transcriptomics-cell-counts.csv |
| Input for RUI2CTpop Workflow: sc-transcriptomics cell counts (with pre-annotated cell counts) | Contains the number of annotated cells in sc-transcriptomics datasets that are part of the input for RUI2CTpop Workflow that have been mapped via CTann tools (see **Box 1**). Pre-annotated cell counts are the number of rows in the cell by gene matrix of the H5AD file. Some cells may not get annotated for various reasons, thus the discrepancy | github.com/x-atlas-consortia/hra-pop/blob/main/output-data/v1.0/reports/universe-ad-hoc/universe-sc-transcriptomics-cell-instance-counts.csv |
| Input for RUI2CTpop Workflow: sc-proteomics cell counts | Contains the number of annotated cells in sc-proteomics datasets that are part of the HRApop Atlas | github.com/x-atlas-consortia/hra-pop/blob/main/output-data/v1.0/reports/universe-ad-hoc/universe-sc-proteomics-cell-counts.csv |
| HRApop Atlas: sc-transcriptomics cell counts | Contains the number of annotated cells in sc-transcriptomics datasets that are part of the HRApop Atlas | github.com/x-atlas-consortia/hra-pop/blob/main/output-data/v1.0/reports/atlas-ad-hoc/atlas-sc-transcriptomics-cell-counts.csv |
| HRApop Atlas: sc-proteomics cell counts | Contains the number of annotated cells in sc-proteomics datasets that are part of the HRApop Atlas | github.com/x-atlas-consortia/hra-pop/blob/main/output-data/v1.0/reports/atlas-ad-hoc/atlas-sc-proteomics-cell-counts.csv |
| HRApop Atlas: Number of extraction sites per AS and intersection volume/percentage of the extraction site (see **Box 1**) | Lists extraction site, organ/AS, intersection volume, AS volume and tissue block volume. | github.com/x-atlas-consortia/hra-pop/blob/main/output-data/v1.0/reports/atlas/table-s5.csv |

bioRxiv preprint doi: https://doi.org/10.1101/2025.08.14.670406; this version posted August 20, 2025. The copyright holder for this preprint (which was not certified by peer review) is the author/funder, who has granted bioRxiv a license to display the preprint in perpetuity. It is made available under aCC-BY 4.0 International license.

| | | |
|---|---|---|
| SPARQL queries | Contains SPARQL queries for all reports generated in in the output data folder on GitHub[125] | github.com/x-atlas-consortia/hra-pop/tree/main/queries |

bioRxiv preprint doi: https://doi.org/10.1101/2025.08.14.670406; this version posted August 20, 2025. The copyright holder for this preprint (which was not certified by peer review) is the author/funder, who has granted bioRxiv a license to display the preprint in perpetuity. It is made available under aCC-BY 4.0 International license.

**Table S4. CT choices.** 26 of the 201 CTs were classified under two different high-level CTs. This table shows the decision process used to select the most appropriate grouping class for each CT.

| CT | Chosen Category | Non-chosen category | Rationale |
|---|---|---|---|
| hematopoietic stem cell | hematopoietic cell | stem cell | Reflects lineage identity central to its biological role |
| neuroendocrine cell | epithelial cell | neural cell | Developmentally and structurally epithelial |
| pancreatic D cell | epithelial cell | neural cell | Developmentally and structurally epithelial |
| intestinal crypt stem cell of large intestine | stem cell | epithelial cell | Stemness prioritized as foundational to epithelial renewal |
| intestinal crypt stem cell of small intestine | stem cell | epithelial cell | Stemness prioritized as foundational to epithelial renewal |
| lung interstitial macrophage | hematopoietic cell | connective tissue cell | Lineage from hematopoietic system prioritized over location |
| pulmonary neuroendocrine cell | epithelial cell | neural cell | Developmentally and structurally epithelial |
| granulocyte monocyte progenitor cell | hematopoietic cell | bone cell | Reflects hematopoietic lineage over location in the bone marrow |
| mesenchymal stem cell of adipose tissue | stem cell | connective tissue cell | Stemness and multipotency prioritized over local connective tissue association. |
| mesenchymal stem cell of abdominal adipose tissue | stem cell | connective tissue cell | Stemness and multipotency prioritized over local connective tissue association. |
| central nervous system macrophage | hematopoietic cell | neural cell | Immune lineage prioritized over CNS location |
| vascular leptomeningeal cell | connective tissue cell | neural cell | Mesenchymal origin and structural connective function prioritized over CNS location |
| mature microglial cell | hematopoietic cell | neural cell | Immune lineage prioritized over CNS location |
| ependymal cell | neural cell | epithelial cell | Derived from neuroectoderm and part of CNS despite epithelial morphology |
| smooth muscle cell of the brain vasculature | muscular cell | neural cell | Reflects muscular identity, over location |
| mesenchymal stem cell | stem cell | connective tissue cell | Stemness and multipotency prioritized over local connective tissue association. |
| basal cell of epidermis | stem cell | epithelial cell | Stemness emphasized as central to epidermal renewal |

bioRxiv preprint doi: https://doi.org/10.1101/2025.08.14.670406; this version posted August 20, 2025. The copyright holder for this preprint (which was not certified by peer review) is the author/funder, who has granted bioRxiv a license to display the preprint in perpetuity. It is made available under aCC-BY 4.0 International license.

| | | | |
|---|---|---|---|
| choroid plexus epithelial cell | neural cell | epithelial cell | Derived from neuroectoderm and part of CNS despite epithelial morphology |
| microglial cell | hematopoietic cell | neural cell | Primitive macrophage origin prioritized over CNS-resident role |
| type D cell of colon | epithelial cell | neural cell | Developmentally and structurally epithelial |
| type EC enteroendocrine cell | epithelial cell | neural cell | Developmentally and structurally epithelial |
| cycling type EC enteroendocrine cell | epithelial cell | neural cell | Developmentally and structurally epithelial |
| type D cell of small intestine | epithelial cell | neural cell | Developmentally and structurally epithelial |
| retinal blood vessel endothelial cell | endothelial cell | neural cell | Endothelial identity and vascular role prioritized over retinal location |
| retinal pigment epithelial cell | epithelial cell | neural cell | Morphology and function as barrier epithelium prioritized over retinal location |
| endosteal cell | bone cell | epithelial cell | Skeletal lineage and role in bone homeostasis prioritized over lining morphology |

bioRxiv preprint doi: https://doi.org/10.1101/2025.08.14.670406; this version posted August 20, 2025. The copyright holder for this preprint (which was not certified by peer review) is the author/funder, who has granted bioRxiv a license to display the preprint in perpetuity. It is made available under aCC-BY 4.0 International license.

**Table S5. CTann settings.** Docker containers with full contexts for the three CTann tools are available on GitHub[136].

| Tool | Version | Code base | Models | Requirements |
|---|---|---|---|---|
| Azimuth | v0.4.6 | R | kidneyref/Kidney_L3/annotation.l3<br>lungref/Lung_v2_finest_level/ann_finest_level<br>heartref/Heart_L2/celltype.l2<br>humancortexref/subclass<br>pancreasref/Pancreas_L1/annotation.l1<br>pbmcref/Human_PBMC_L2/celltype.l2<br>bonemarrowref/Bone_marrow_L2/celltype.l2<br>adiposeref/Adipose_L2/celltype.l2 | anndata==0.9.1<br>contourpy==1.0.7<br>cycler==0.11.0<br>fonttools==4.39.4<br>h5py==3.8.0<br>importlib-metadata==6.6.0<br>importlib-resources==5.12.0<br>joblib==1.2.0<br>kiwisolver==1.4.4<br>llvmlite==0.40.1rc1<br>matplotlib==3.7.1<br>natsort==8.3.1<br>networkx==3.1<br>numba==0.57.0<br>numpy==1.24.3<br>packaging==23.1<br>pandas==2.0.2<br>patsy==0.5.3<br>Pillow==9.5.0<br>pynndescent==0.5.10<br>pyparsing==3.0.9<br>python-dateutil==2.8.2<br>pytz==2023.3<br>scanpy==1.9.3<br>scikit-learn==1.2.2<br>scipy==1.10.1<br>seaborn==0.12.2<br>session-info==1.0.0<br>six==1.16.0<br>statsmodels==0.14.0<br>stdlib-list==0.8.0<br>threadpoolctl==3.1.0<br>tqdm==4.65.0<br>tzdata==2023.3<br>umap-learn==0.5.3<br>zipp==3.15.0 |
| CellTypist | v1.6 | Python | Human_Lung_Atlas.pkl<br>Alternative: Cells_Lung_Airway.pkl<br>Adult_Human_Skin.pkl<br>Adult_Human_PancreaticIslet.pkl<br>Adult_Human_PancreaticIslet.pkl<br>Healthy_Adult_Heart.pkl<br>Healthy_Human_Liver.pkl<br>Human_AdultAged_Hippocampus.pkl<br>Human_Longitudinal_Hippocampus.pkl<br>Cells_Intestinal_Tract.pkl | anndata==0.9.2<br>celltypist==1.6.1<br>certifi==2023.7.22<br>charset-normalizer==3.3.1<br>click==8.1.7<br>contourpy==1.1.1<br>cycler==0.12.1<br>et-xmlfile==1.1.0<br>fonttools==4.43.1<br>h5py==3.10.0<br>idna==3.4<br>igraph==0.10.8<br>joblib==1.3.2<br>kiwisolver==1.4.5<br>leidenalg==0.10.1<br>llvmlite==0.41.1<br>matplotlib==3.8.0<br>natsort==8.4.0<br>networkx==3.2 |

bioRxiv preprint doi: https://doi.org/10.1101/2025.08.14.670406; this version posted August 20, 2025. The copyright holder for this preprint (which was not certified by peer review) is the author/funder, who has granted bioRxiv a license to display the preprint in perpetuity. It is made available under aCC-BY 4.0 International license.

| | | | | |
|---|---|---|---|---|
| | | | | numba==0.58.1<br>numpy==1.24.4<br>openpyxl==3.1.2<br>packaging==23.2<br>pandas==2.0.3<br>patsy==0.5.3<br>Pillow==10.1.0<br>pynndescent==0.5.10<br>pyparsing==3.1.1<br>python-dateutil==2.8.2<br>pytz==2023.3.post1<br>requests==2.31.0<br>scanpy==1.9.5<br>scikit-learn==1.3.2<br>scipy==1.11.3<br>seaborn==0.13.0<br>session-info==1.0.0<br>six==1.16.0<br>statsmodels==0.14.0<br>stdlib-list==0.9.0<br>texttable==1.7.0<br>threadpoolctl==3.2.0<br>tqdm==4.66.1<br>tzdata==2023.3<br>umap-learn==0.5.4<br>urllib3==2.0.7 |
| popV | See commit github.com/YosefLab/popV/tree/2d29c9a290d2015ec65ef0ef9f0e6b6d2277e7bb | Python | model: Bladder<br>organ_level:urinary bladder<br><br>model: Blood<br>organ_level: blood<br><br>model: Bone_Marrow<br>organ_level: bone marrow<br><br>model: Eye<br>organ_level: eye<br><br>model: Fat<br>organ_level: adipose tissue<br><br>model: Large_Intestine<br>organ_level: large intestine<br><br>model: Liver<br>organ_level: liver<br><br>model: Lung<br>organ_level: lung<br><br>model: Lymph_Node<br>organ_level: mesenteric lymph node<br><br>model: Mammary<br>organ_level: mammary gland<br><br>model: Pancreas<br>organ_level: pancreas | absl-py==2.1.0<br>aiohappyeyeballs==2.4.0<br>aiohttp==3.10.5<br>aiosignal==1.3.1<br>anndata==0.10.9<br>annoy==1.17.3<br>array_api_compat==1.8<br>astunparse==1.6.3<br>attrs==24.2.0<br>bbknn==1.6.0<br>beautifulsoup4==4.12.3<br>celltypist==1.6.3<br>certifi==2024.8.30<br>charset-normalizer==3.3.2<br>chex==0.1.86<br>click==8.1.7<br>contextlib2==21.6.0<br>contourpy==1.3.0<br>cycler==0.12.1<br>Cython==3.0.11<br>docrep==0.3.2<br>et-xmlfile==1.1.0<br>etils==1.9.4<br>fbpca==1.0<br>filelock==3.15.4<br>flatbuffers==24.3.25<br>flax==0.9.0<br>fonttools==4.53.1<br>frozenlist==1.4.1<br>fsspec==2024.9.0<br>gast==0.6.0<br>gdown==5.2.0<br>geosketch==1.2<br>google-pasta==0.2.0 |

bioRxiv preprint doi: https://doi.org/10.1101/2025.08.14.670406; this version posted August 20, 2025. The copyright holder for this preprint (which was not certified by peer review) is the author/funder, who has granted bioRxiv a license to display the preprint in perpetuity. It is made available under aCC-BY 4.0 International license.

| | | | | |
|---|---|---|---|---|
| | | | model: Prostate<br>Organ_level: prostate gland<br><br>model: Salivary Gland<br><br>model: Skin<br>Organ_level: skin<br><br>model: Small_Intestine<br>organ_level: small intestine<br><br>model: Spleen<br>organ_level: spleen<br><br>model: Thymus<br>organ_level: thymus<br><br>model: Tongue<br><br>model: Trachea<br>organ_level: trachea<br><br>model: Uterus<br>organ_level: uterus<br><br>model: Vasculature<br>organ_level: blood vasculature | grpcio==1.66.1<br>h5py==3.11.0<br>harmony-pytorch==0.1.8<br>huggingface-hub==0.24.6<br>humanize==4.10.0<br>idna==3.8<br>igraph==0.11.6<br>importlib_resources==6.4.4<br>intervaltree==3.1.0<br>jax==0.4.31<br>jaxlib==0.4.31<br>Jinja2==3.1.4<br>joblib==1.4.2<br>keras==3.5.0<br>kiwisolver==1.4.7<br>legacy-api-wrap==1.4<br>leidenalg==0.10.2<br>libclang==18.1.1<br>lightning==2.1.4<br>lightning-utilities==0.11.7<br>llvmlite==0.43.0<br>Markdown==3.7<br>markdown-it-py==3.0.0<br>MarkupSafe==2.1.5<br>matplotlib==3.9.2<br>mdurl==0.1.2<br>ml-dtypes==0.4.0<br>ml_collections==0.1.1<br>mpmath==1.3.0<br>msgpack==1.0.8<br>mudata==0.3.1<br>multidict==6.0.5<br>multipledispatch==1.0.0<br>namex==0.0.8<br>natsort==8.4.0<br>nest-asyncio==1.6.0<br>networkx==3.3<br>numba==0.60.0<br>numpy==1.26.4<br>numpyro==0.15.2<br>nvidia-cublas-cu12==12.1.3.1<br>nvidia-cuda-cupti-cu12==12.1.105<br>nvidia-cuda-nvrtc-cu12==12.1.105<br>nvidia-cuda-runtime-cu12==12.1.105<br>nvidia-cudnn-cu12==9.1.0.70<br>nvidia-cufft-cu12==11.0.2.54<br>nvidia-curand-cu12==10.3.2.106<br>nvidia-cusolver-cu12==11.4.5.107<br>nvidia-cusparse-cu12==12.1.0.106<br>nvidia-nccl-cu12==2.20.5<br>nvidia-nvjitlink-cu12==12.6.68<br>nvidia-nvtx-cu12==12.1.105<br>obonet==1.1.0<br>OnClass==1.3<br>openpyxl==3.1.5<br>opt-einsum==3.3.0<br>optax==0.2.3<br>optree==0.12.1<br>orbax-checkpoint==0.6.1<br>packaging==24.1 |

bioRxiv preprint doi: https://doi.org/10.1101/2025.08.14.670406; this version posted August 20, 2025. The copyright holder for this preprint (which was not certified by peer review) is the author/funder, who has granted bioRxiv a license to display the preprint in perpetuity. It is made available under aCC-BY 4.0 International license.

| | | | | |
|---|---|---|---|---|
| | | | | pandas==1.5.3<br>patsy==0.5.6<br>pillow==10.4.0<br>PopV @ git+github.com/czbiohub/PopV@2d29c9a290d2015ec65ef0ef9f0e6b6d2277e7bb<br>protobuf==4.25.4<br>psutil==6.0.0<br>Pygments==2.18.0<br>pynndescent==0.5.13<br>pyparsing==3.1.4<br>pyro-api==0.1.2<br>pyro-ppl==1.9.1<br>PySocks==1.7.1<br>python-dateutil==2.9.0.post0<br>pytorch-lightning==2.4.0<br>pytz==2024.1<br>PyYAML==6.0.2<br>regex==2024.7.24<br>requests==2.32.3<br>rich==13.8.0<br>safetensors==0.4.5<br>scanorama==1.7.4<br>scanpy==1.10.2<br>scikit-learn==1.1.3<br>scikit-misc==0.5.1<br>scipy==1.14.1<br>scvi-tools==1.1.6<br>seaborn==0.13.2<br>sentence-transformers==3.0.1<br>session_info==1.0.0<br>six==1.16.0<br>sortedcontainers==2.4.0<br>soupsieve==2.6<br>statsmodels==0.14.2<br>stdlib-list==0.10.0<br>sympy==1.13.2<br>tensorboard==2.17.1<br>tensorboard-data-server==0.7.2<br>tensorflow==2.17.0<br>tensorflow-io-gcs-filesystem==0.37.1<br>tensorstore==0.1.65<br>termcolor==2.4.0<br>texttable==1.7.0<br>threadpoolctl==3.5.0<br>tokenizers==0.19.1<br>toolz==0.12.1<br>torch==2.4.1<br>torchmetrics==1.4.1<br>tqdm==4.66.5<br>transformers==4.44.2<br>triton==3.0.0<br>typing_extensions==4.12.2<br>umap-learn==0.5.6<br>urllib3==2.2.2<br>Werkzeug==3.0.4<br>wget==3.2<br>wrapt==1.16.0<br>yarl==1.9.11 |

bioRxiv preprint doi: https://doi.org/10.1101/2025.08.14.670406; this version posted August 20, 2025. The copyright holder for this preprint (which was not certified by peer review) is the author/funder, who has granted bioRxiv a license to display the preprint in perpetuity. It is made available under aCC-BY 4.0 International license.

| | | | | |
|---|---|---|---|---|
| | | | | zenodo-get==1.5.1<br>zipp==3.20.1 |
